# Adding colour-realistic video images to audio playbacks increases stimulus engagement but does not enhance vocal learning in zebra finches

**DOI:** 10.1007/s10071-021-01547-8

**Published:** 2021-08-17

**Authors:** Judith M. Varkevisser, Ralph Simon, Ezequiel Mendoza, Martin How, Idse van Hijlkema, Rozanda Jin, Qiaoyi Liang, Constance Scharff, Wouter H. Halfwerk, Katharina Riebel

**Affiliations:** 1 Institute of Biology Leiden, Leiden University, Leiden, The Netherlands; 2 Department of Ecological Science, VU University Amsterdam, Amsterdam, The Netherlands; 3 Nuremberg Zoo, Nuremberg, Germany; 4 Institut für Biologie, Freie Universität Berlin, Berlin, Germany; 5 School of Biological Sciences, University of Bristol, Bristol, UK; 6 Evolution of Sensory Systems, Max Planck Institute for Ornithology, Seewiesen, Germany

**Keywords:** Bird song, Vocal development, Video tutors, Multimodal communication

## Abstract

Bird song and human speech are learned early in life and for both cases engagement with live social tutors generally leads to better learning outcomes than passive audio-only exposure. Real-world tutor–tutee relations are normally not uni- but multimodal and observations suggest that visual cues related to sound production might enhance vocal learning. We tested this hypothesis by pairing appropriate, colour-realistic, high frame-rate videos of a singing adult male zebra finch tutor with song playbacks and presenting these stimuli to juvenile zebra finches (*Taeniopygia guttata*). Juveniles exposed to song playbacks combined with video presentation of a singing bird approached the stimulus more often and spent more time close to it than juveniles exposed to audio playback only or audio playback combined with pixelated and time-reversed videos. However, higher engagement with the realistic audio–visual stimuli was not predictive of better song learning. Thus, although multimodality increased stimulus engagement and biologically relevant video content was more salient than colour and movement equivalent videos, the higher engagement with the realistic audio–visual stimuli did not lead to enhanced vocal learning. Whether the lack of three-dimensionality of a video tutor and/or the lack of meaningful social interaction make them less suitable for facilitating song learning than audio–visual exposure to a live tutor remains to be tested.

**Supplementary Information:**

The online version contains supplementary material available at 10.1007/s10071-021-01547-8.

## Introduction

Bird song is one of the best-studied animal examples of vocally learned signalling (Catchpole and Slater 1995) and it is often used as a model system for human speech acquisition, because of the many similarities between human speech and bird song (Doupe and Kuhl 1999; Bolhuis et al. 2010). One of the open research questions in the study of both speech and bird song development is whether, and to what extent, exposure to the visual cues accompanying the production of vocalizations, such as lip movements in speech and beak movements in bird song, plays a role in vocal development (speech: Kuhl and Meltzoff 1982; Lewkowicz and Hansen-Tift 2012; Teinonen et al. 2008; Tenenbaum et al. 2015, birdsong: Beecher and Burt 2004; Derégnaucourt 2011; Slater et al. 1988). Given the well-established experimental tutoring paradigms, bird song offers a system in which the effect of visual cues on the vocal learning process can be studied experimentally (Doupe and Kuhl 1999; Brainard and Doupe 2002; Goldstein et al. 2003).

In the study of bird song learning, experimental tape-tutoring has been crucial. Instead of learning from a bird that is physically present, young birds are tutored by playing back pre-recorded conspecific song via loudspeakers, either under operant control of the juvenile bird or passively (Derégnaucourt 2011). These methods allow researchers control over the quantity, quality and timing of song exposure. This high level of experimental and stimulus control has greatly contributed to understanding vocal learning processes (Catchpole and Slater 1995; Derégnaucourt 2011). Not all songbird species, however, learn as well from a tape tutor as from a live conspecific (reviewed in Baptista and Gaunt 1997; Soma 2011). Many researchers have argued that this is because social interaction with a tutor is important for song learning (e.g. see Baptista and Petrinovich 1986; Slater et al. 1988; Catchpole and Slater 1995; Carouso-Peck et al. 2020). However, tape and live tutors differ in more aspects than sociality. For example, bird song, like much animal communication, is multimodal, offering simultaneous information from several modalities (Partan and Marler 1999; Higham and Hebets 2013; Halfwerk et al. 2019). Bird song production is accompanied by visual components, such as beak, head, throat and body movements. Multimodal signals are often easier detected and remembered by receivers than unimodal signals (reviewed in Rowe 1999) and might thus be beneficial to learning. In line with this, improved learning of paired auditory–visual stimuli has been demonstrated in several bird species and contexts, for example in the context of filial imprinting (van Kampen and Bolhuis 1991, 1993) or song learning (e.g. in nightingales, *Luscinia megarhynchos*, Hultsch et al. 1999). However, the difference between multi- and unimodal tutoring has rarely been considered in the discussion on why several bird species learn better from live- than from tape tutors (Nelson 1997; Baptista and Gaunt 1997; Soma 2011).

One of the songbird species often cited for learning poorly from audio playbacks is the zebra finch (*Taeniopygia guttata*), an important animal model to study vocal learning (Griffith and Buchanan 2010; Mello 2014). Zebra finches learn better from a live tutor than when passively exposed to audio-only presentation of tutor song (Eales 1989; Derégnaucourt et al. 2013; Chen et al. 2016). The most favoured hypothesis regarding these differences is that social interactions with a tutor increase the salience of the tutor song (Chen et al. 2016; Derégnaucourt et al. 2013; Slater et al. 1988). However, social and tape tutors also differ in non-social aspects: tape-tutoring is often more stereotyped than a live tutor, shows no circadian activity patterns, is less or not interactive and is often non-contingent on tutee behaviour (for discussion see Nelson 1997). The effect of contingencies on song learning has seen some experimental testing in zebra finches, but with mixed results regarding whether they facilitate song learning from playback and whether similar learning outcomes can be attained with behaviour contingent playback as with live tutoring (ten Cate 1991; Adret 1993; Houx and ten Cate 1999a; Phan et al. 2006; Derégnaucourt et al. 2013). There is, however, yet an additional systematic difference that studies investigating social versus non-social tutoring have not controlled for, namely the multi- versus unimodal presentation of song in live compared to classic tape-tutoring paradigms. In this study, we aim to specifically test whether multimodal exposure (rather than social interaction) to a tutor might improve learning and could thus (partly) explain the differences in learning from tape and live tutors. To do so, a method is required that allows investigating whether song learning from passive song playback is improved by simultaneous visual exposure to the singing tutor when, akin to tape-tutoring, tutees cannot also socially interact with the song tutor.

This study follows up on earlier pioneering experiments that added visual stimuli right before, during or after the presentation of tutor song and found no improvement of learning with the added visual stimuli (Bolhuis et al. 1999; Houx and ten Cate 1999b). These studies used non-moving taxidermic mounts of male zebra finches as visual stimuli, which might have been suboptimal because they were stationary (Bolhuis et al. 1999). Interestingly, painted plaster images of female conspecifics were sufficient to stimulate adult males to sing more than when alone (Bischof et al. 1981), suggesting that the degree of naturalistic visual stimulation necessary for song learning in juveniles and song production in adults might differ.

Videos provide moving images, but when using videos in animal research, it should be taken into consideration that standard video systems are designed for human visual perception. This aspect was until recently rarely controlled and adjusted for during video stimulus preparation and presentation to animals that often have different colour and movement perception (Chouinard-Thuly et al. 2017). Birds have a higher flicker-fusion frequency and different colour, brightness and depth perception than humans (Cuthill et al. 2000; Fleishman and Endler 2000; Oliveira et al. 2000). It is unclear, however, how much deviation from naturalistic colour and movement fluidity is still acceptable to birds. Human vision-adapted videos can trigger natural behaviour in zebra finches, such as copying food choices from demonstrators via live streaming videos (Guillette and Healy 2016) or courtship singing by males towards females on video screens (Ikebuchi and Okanoya 1999; Galoch and Bischof 2007; James et al. 2019) and presenting a video of a female conspecific contingent with immature song production by juvenile male zebra finches improves song learning (Carouso-Peck and Goldstein 2019). Importantly, zebra finches do react differently to a video than a live presentation of particular stimuli (Ikebuchi and Okanoya 1999; Swaddle et al. 2006; Guillette and Healy 2019; James et al. 2019). Zebra finches tutored with a passive or operant video tutor copied song poorly (Adret 1997). Adret (1997) speculated that the poor sound quality of the TV monitor loudspeakers used for playbacks might have been responsible for the poor learning and other authors later wondered whether the low flicker frequency of the monitor in this experiment was suboptimal (Derégnaucourt 2011). Neither factor has been systematically tested so far in the context of song learning. High-fidelity audio–video playbacks could open a window into investigating the potential role of multimodal cues in song learning, a potential confound of ‘social’ tutoring not controlled in classic audio-only playback studies. Deshpande et al. (2014) conducted a study in which juvenile zebra finches had operant control over either just audio or audio–visual (simultaneous or staggered audio and video) playback of song. In this study, only the groups tutored with simultaneous audio–visual playback or with staggered playback where audio preceded video showed significant song learning compared to birds without tutoring. Song learning in all birds was poor, possibly because the video was sub-optimally adjusted to avian vision (e.g. no colour adjustments) or because of the very limited amount of song exposure that birds received (only 75 s in total over the sensitive period for song learning). In addition, only one tutor video was used, so any unintended cue or flaw in this particular video may have unduly influenced the results. Technical advancement and increased insights into avian vision allow addressing several of the potential issues with stimulus quality discussed above and formulated in a recent consensus on the usage of video stimuli in animal research (Chouinard-Thuly et al. 2017). A recurrent, neglected issue in this context is how the frame of the presented video relates to the study species’ speed of vision. Neglecting such aspects can affect animals’ responses, as has been demonstrated for social responses of pigeons, *Columbia livia,* towards video stimuli (Ware et al. 2015). In the present study, we therefore made use of recent technical, empirical and theoretical advancements to produce videos of multiple tutors. We recorded them with high frame rates (120 fps) to accommodate the higher temporal resolution of zebra finch vision and videos were displayed on gaming monitors with high refresh rates (120 Hz), which, in combination with the high frame rates of the video itself, should make the movements in the videos look smooth to the birds. We also adjusted the colours of our videos following the ‘colour-realistic imagery’ technique (Tedore and Johnsen 2017), to mimic as closely as possible the animals’ colour perceptual experience of a real conspecific. Combining these videos with high-quality sound recordings, enabled us to present auditory and visual information linked in real-time (or experimentally dissociated) to zebra finch tutees, thus controlling for currently known potential sources of artefacts (Chouinard-Thuly et al. 2017).

In the current study, tutees were exposed to either audio playback only or to song playbacks accompanied by colour-realistic videos of the singing tutor or in a control condition by the same colour-realistic, but now pixelated and reversed versions of the video stimuli. If accurate rhythmic correspondence between the beak, head, and throat movements and the song facilitates song learning, the birds receiving video presentations of the tutor together with audio playback should show improved song learning. It is also possible that any moving visual stimulus presented together with the song would facilitate song learning. For instance, the detectability of a signal can be positively affected if it is presented together with an additional stimulus in another sensory modality, possibly by drawing the receiver’s attention to the signal (Feenders et al. 2017; reviewed in Rowe 1999). We therefore also included a group of tutees exposed to videos created by pixelating the frames of the original videos before playing them back in reversed order. This created videos of comparable complexity in colours and movements without presenting a video image of a bird and without direct rhythmic correspondence between the song and the video. To prevent possible effects that social isolation might have on song learning, which in tape versus live tutoring is a rarely addressed confound (Varkevisser et al. in prep.), we decided to not house the tutees solitarily, as was usually the case in previous zebra finch tape-tutoring studies (e.g. Bolhuis et al. 1999; Derégnaucourt et al. 2013; Houx and ten Cate 1999a, b), but together with an age-matched female companion. Being housed with a companion will likely be beneficial for welfare and can potentially motivate a bird to sing (Jesse and Riebel 2012), thereby creating a better comparison with a situation where a live tutor is present. As all female companions, like the male tutees, came from families where the father had been removed before the onset of the sensitive phase for song learning, females might reinforce singing in males (as in the natural nest), but any influence they might have will be unspecific with regard to song content.

By thus keeping the social environment the same, but varying whether song presentation was accompanied by visual stimulation (song unspecific versus song specific), we created an experimental situation to test the hypothesis that visual stimulation in addition to auditory song exposure facilitates song learning. If this were the case, then all video tutored birds should learn better compared to birds experiencing only unimodal auditory song exposure. In addition, the video groups might differ from each other in learning outcomes if visual exposure to the specific movements accompanying song production, e.g. song-related beak and body movements, had greater salience in this context than equally colourful and equally animated, but unspecific visual exposure. This expectation was based on the human literature where such sound-specific motor gestures attract the attention of infants more than unspecific gestures (Kuhl and Meltzoff 1982; Patterson and Werker 1999), but also on increased insights from the animal literature showing effects of correctly synchronised visual and acoustic information on perceptual salience (e.g. Taylor et al. 2011; Rȩk 2018). We thus expected the tutor videos with the synchronous auditory–visual information to lead to better song learning than the pixelated and reversed videos.

## Methods

### Subjects and housing

We used 44 juvenile males and 44 juvenile females from the domesticated wild-type zebra finches breeding colony at Leiden University. Birds were raised and housed in breeding cages (100 × 50 × 40 cm) with their parents and siblings until 20 days post-hatching (dph, calculated as days from the median hatching day within a nest), when the father was removed. Subjects stayed with their mother and siblings from 20 to 35 dph in their home cage. All breeding cages were located in a large breeding room with multiple pairs breeding in two long stacks of cages along the two long walls. At all times, other birds could be heard and the birds 2.40 m across on the opposite side of the aisle could also be seen. When subjects reached 35 dph, they were moved in dyads consisting of a young male and an unrelated young female into sound-attenuated chambers (125 × 300 × 240 cm) for song tutoring (details below) until they reached 65 dph, when they were moved to a recording cage (see below). After recording at 65 dph, the dyads were housed in separate cages (150 × 40 × 50 cm) located in a room with multiple birds, until song of the male tutees was recorded after 100 dph (see below).

Throughout, birds were housed on a 13.5/10.5 light/dark cycle (with 30 min dusk and dawn simulations), at 20–22 °C and 45–65% humidity. Birds had ad libitum access to a commercial tropical seed mixture (Beyers, Belgium), cuttlebone, grit and drinking water. This diet was supplemented three times a week with hardboiled eggs and once a week with germinated tropical seeds, vegetables and fruit.

### Song tutoring

For this study, a song was defined as one or several motifs separated from other sounds by more than 2 s of silence or when a motif was starting with additional introductory notes (Sossinka and Böhner 1980). Motifs were defined as the repeated syllable sequence in a song, and syllables as sounds separated from other sounds by at least 5 ms of silence.

**Fig. 1:**
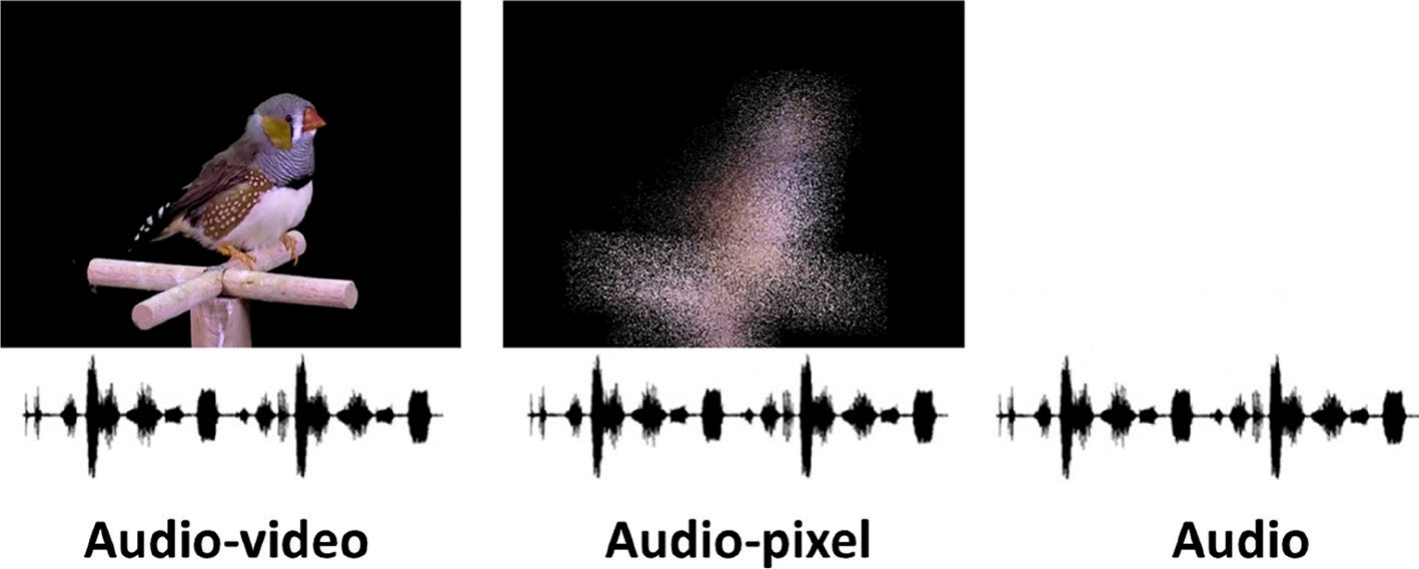
Overview of the different tutoring treatments in this study. The audio–video treatment consisted of a synchronous sound and video exposure (120 fps video, sound and beak movements aligned, for an example see Online Resource 1); the audio-pixel treatment consisted of the same song and the same video, but the video was pixelated and played back in reversed order (for an example see Online Resource 2) and in the audio treatment only the audio channel of the song was played back

A male–female tutee dyad was exposed to one of three different tutoring treatments (Fig. 1): (1) song only playback (“Audio”), (2) song playback combined with a time-aligned video of the tutor singing (“Audio–video”) or (3) song playback combined with a pixelated version of the same video and with the individual frames of the video played back in reversed order (“Audio-pixel”).

We used song from 12 different tutors. The same tutor song was presented to three tutee dyads, each in a different tutoring treatment (audio, audio–video and audio-pixel). Tutees exposed to the same tutor song were tutored simultaneously and will be referred to as one ‘tutor group’. We raised 12 tutor groups with these three treatments. Due to a technical delay in another experiment, additional young birds could be tutored and post hoc, we raised four additional tutor groups. In these four groups, we only included the audio–video and audio-pixel treatment to increase the statistical power for the pairwise comparisons in the sub-question as to whether the quality of the video material affected learning. For these groups, we used four tutors that had previously been used as tutors for other groups. Within one tutor group, wherever possible, all males and all females originated from the same nest (all 3 male siblings: 8/12 tutor groups; 2 siblings and 1 additional male: 3/12 tutor groups; 3 unrelated males: 1/12 tutor groups; all 3 female siblings: 11/12 tutor groups; 2 siblings and 1 additional female: 1/12 tutor groups). Tutoring took place between 35 and 65 days post hatching. Tutor songs were presented in daily tutoring sessions following one of three different tutoring schedules (see Table 1 for details). For each tutor, per treatment, three different stimuli were made which were played back in random order throughout the day. It is currently unclear how often a tutee should hear a tutor song to optimally learn it. Some studies suggested that a high amount of song exposure might negatively affect zebra finch song learning (Tchernichovski et al. 1999; Tchernichovski and Mitra 2002; Chen et al. 2016). However, previous passive play-back studies have found a low degree of tutor song copying using exposure frequencies ranging from 20 (Derégnaucourt et al. 2013) to approximately 250 songs per day (Bolhuis et al. 1999; Houx and ten Cate 1999b). Even less is known about how much a tutee should be exposed to a video tutor, but given the limitations of producing sufficient high-quality videos and a potential effect of overexposure, we decided to first offer limited song exposure to the first three tutor groups. These groups (i.e. 3 × 3 male tutees, in the audio–video, audio-pixel and audio condition) received three tutoring sessions daily with 10 songs played during each session (schedule 1). We made daily observations of how tutees responded to the stimulus presentation (through the one-way mirrors in the doors of the sound-attenuated chambers). At the end of the song tutoring period, tutees in these groups still responded to the stimulus presentation by approaching the loudspeakers and thus did not seem to lose interest in the stimuli over time. We also observed that it took a while before the birds reached the best position to see the videos, which they left again during the inter-song intervals. This sometimes meant they only saw part of the video. We thus decided to increase the number of tutoring sessions and the amount of song presented per session and to shorten the inter-song intervals. The next nine tutor groups thus received four tutoring sessions daily with 12 songs per session (schedule 2). As the tutees still seemed to remain interested in the stimuli throughout the experiment, we decided to increase exposure even further during the third schedule. Given the exploratory nature of the study, using several exposure frequencies seemed safest to detect potential effects of exposure frequency that could then inspire future studies and also safest to avoid both floor and ceiling effects from exposure frequency. The last four tutor groups therefore received eight tutoring sessions daily with 24 songs per session (schedule 3), reaching daily song exposures of 192 songs and an average of 768 motifs, which falls into the range of daily song output observed in adult males housed socially (range between 0 and 1262 motifs, average ± SD: 395 ± 362 motifs; Jesse and Riebel 2012, range between 0 and 891 motifs, average ± SD: 237 ± 208 motifs; Böhner 1983). In all schedules and for all treatments, the first session began at 08:15, half an hour after the lights went on in the room and every tutoring session started with the audio-only presentation of three introductory notes of the tutor followed by 1 s of silence. After this, one of three different videos and/or songs of the same tutor was presented. After the stimulus presentations, the screens went back to black.

**Table 1:** Description of the different tutoring schedules used in this study

Schedule	# daily tutoring sessions	Daily tutoring times	# songs/session	# songs/day	Inter-song interval	*N* groups
1	3	8:15 12:15, 16:15	10	30	Fixed 1 min	3
2	4	8:15, 10:15 12:15, 16:15	12	48	Variable range 2–6 s^c^	9
3^a^	8	8:15, 8:45, 9:15, 10:15 12:15, 13:30, 14:45, 16:15	24	192	Variable range 2–6 s	4^b^

^a^With this schedule, no birds were tutored in the Audio condition

^b^All tutor groups had a different tutor song, but these four groups received the songs of 4 of the tutors used in schedule 2

^c^The playback program used random inter-song intervals in the given range

### Stimulus preparation

#### Audio and video recordings

Stimuli consisted of audio and video recordings of undirected song of 12 adult male zebra finches from the colony (3 songs per bird, 36 songs in total). All songs were recorded in an identical manner and using the same equipment: a male was placed singly in a recording cage (76 × 45 × 45 cm) placed on a table in a sound-attenuated room in the afternoon of the day before recording for acclimation. The next morning, during the time of highest singing activity after lights on, the male was recorded between 08:00 and 11:00, or until we had recorded three songs. After this, the male was returned to its home cage. The recording cage had a clear Plexiglas window in the middle of the front side of the cage. A single cross-shaped perch was placed in the middle of the cage so that the bird would always be in focus of the camera. The back side of the cage was covered with a black cloth so that the videos had a black background, because this gave the best contrast between the background and the stimulus bird. LED video lights (DV-216VC, FalconEyes, Hong Kong) were projected on the perch from the rear above and the left and right front sides. Audio recordings were made with a Sennheiser MKH40 microphone (Wedemark, Germany), hanging 50 cm above the perch in the recording cage, connected to a TASCAM DR-100MKiii recorder (TEAC Corp., Los Angeles, USA). Audio was recorded with a sampling rate of 96 kHz and 16-bit resolution. Video recordings were made with a Casio high-speed camera (EX-ZR3600, 120 fps, 12× optical zoom, Tokyo, Japan) through Plexiglas in the door of the sound-attenuated room. A signal bell (70027 Heidemann, Willich, Germany), which was sound-attenuated to not disturb the birds was attached to the front side of the recording cage above the Plexiglas window and could be triggered from outside the sound-attenuated room. The bell produced a short, impulse like audio signal and it was clearly visible on the video when the clapper touched the bell, which was later used to synchronize the audio and video recordings during stimulus preparations. The camera could record 120 fps videos up to 12 min and at the start of each recording, we triggered the bell. Audio files were filtered with a band-stop filter from 0 to 420 Hz using Praat (version 6.0.19, Boersma and Weenink 2018). Audio and video files were synchronized with Vegas Pro (version 14.0, Magix, Berlin, Germany).

For each male, three songs with introductory notes followed by 3 to 5 motifs were cut out of the recordings (mean song duration ± SD = 4.2 ± 1.2 s, mean number of motif repetitions ± SD = 3.9 ± 0.8).

#### Colour adjustments of the videos

Commercially available RGB displays are made for human vision, and their three phosphors (Red, Green, Blue) match the sensitivity of human cones (560 nm, 530 nm and 420 nm, Solomon and Lennie 2007). Zebra finches, like other birds, are tetrachromatic with four cone types with wavelength sensitivities of 567 nm, 502 nm, 429 nm, and 360–380 nm. Birds thus have a wider visual spectrum (approximately 320–700 nm, incl. UV) than humans (approximately 400–700 nm). This means images or videos displayed on standard LCD screens that emulate human perception of colour rather than the true light reflectance of objects, video playbacks on RGB screens will not provide the true colours to the birds. There is however a method known as *colour-realistic imagery* which allows to colour-correct images displayed on RGB screens (Tedore and Johnsen (2017) to match the colour perception system of a non-human observer as closely as possible. To calculate the correction factors, we needed as input: the colour spectra of the plumage of zebra finches; the sensitivity of their photoreceptors [measured previously by Bowmaker et al. (1997)]; and the output of the phosphors of the experimental RGB displays. As it is not possible to display UV light with monitors, we neglected the UV component and only corrected the red, green and blue channel.

#### Measurements of zebra finch plumage radiance and video screen irradiance

Most zebra finch colour patches are either black, white or grey and they do not need colour correction (or colour correction would only lead to minimal changes), therefore we focused on the three main coloured patches: the red beak, the orange/red cheeks and the brownish lateral patterns beneath the wings. We measured these patches for 6 male zebra finches, using dead birds that were directly frozen after they had been sacrificed for other purposes. For each bird, we took six measurements of the relative radiance of each colour patch with a Flame spectrometer [QR400-7-SR-BX reflection probe and a DH-2000-BAL balanced UV–VIS light source, spectralon white standard, all from Ocean Insight (Orlando, FL, USA)]. We then measured the absolute radiance of the gaming monitors (VG248QE, ASUS, Taipei, Taiwan) to be used to display our stimuli. We used a calibrated light source (HL-3P-CAL) and a 400 um Premium Fiber (QP400-2-VIS-BX), both from Ocean Insight (Orlando, FL, USA) to calibrate the spectrometer. To ensure that the fibre did not move between measurements of the different phosphors, we clamped the bare fibre firmly in front of the screens. We displayed red, green or blue phosphors by setting the measured phosphor value to a middle magnitude 128 and all other phosphors to zero. Measured radiance values were converted to quantal units, see Appendix, Fig. 8 for the results.

#### Generation of colour-adjusted video stimuli

With the zebra finch plumage colour spectra, the birds’ photoreceptor sensitivities and the output of the phosphors of the screens, we could calculate correction factors using a Matlab [R2019a, Mathworks, Natick, Massachusetts, USA—script provided by Tedore and Johnsen (2017)]. We then colour-corrected the single frames of the videos in Photoshop CC (Adobe Inc., Mountain View, California, USA) using the ‘Replace Color’ function (Image > Adjustments > Replace Color) for the different colour patches. For an example of a colour-corrected frame, see Appendix, Fig. 9. We selected the patch with the eyedropper tool, adjusted the selection threshold in a way the whole patch was chosen and not many other parts of the bird were selected and then adjusted using the correction factor values for the respective patch. We used Photoshop droplets to batch process all colour patches and frames. We also created pixelated videos using the Photoshop displacement filter (Filter > Distort > Displace) and used random pixels as displacement map (see Appendix, Fig. 10). The colour-corrected frames were then imported in Vegas Pro software to create a video with 119.88 fps. The frames were placed in chronological order for the audio–video condition and to avoid any rhythmical visual information, in reversed order for the audio-pixel condition. The audio file was then added to the video in Vegas Pro. All generated stimuli were exported as mp4 files (Audio: 448 Kbps, 96 kHz, 16 Bit, AAC, Video: 640 × 480 Progressive, YUV, 50 Mbps). After creating these stimuli, we played them back through the loudspeaker above the experimental arena (see below) and recorded them with a microphone (MKH40, Sennheiser, Wedemark, Germany) positioned inside the cage. Using Praat software, we visually compared the power spectra (Fast Fourier transform) of these recordings with the power spectra of the original stimuli and did not observe any systematic differences (see Appendix, Fig. 11 for an example).

### Experimental arena

The experimental arena consisted of a cage (70 × 60 × 45 cm, see Fig. 3) with four sides of wire mesh in the audio-only condition and three sides of wire mesh and one side of black plastic in the other two conditions. A window (20 × 15 cm) was cut out of the plastic and the experimental monitor (VG248QE, ASUS, Taipei, Taiwan) placed directly behind it. To ensure reproducible luminance and colour representation for all screens, we calibrated the screens before every tutoring round. For calibration, we used a X-Rite i1 Display Studio (Danaher Corp., Grand Rapids, USA) and the program iProfiler with the following settings: White Point CIE Illuminant D65, Luminace 120 cd/m^2^, Tone Response Curve: sRGB. The screen was connected to an Intel NUC computer (NUC7i3BNK, Intel Corporation, California, USA) which controlled stimulus presentation by a custom-made (by one of us—RS) LabView program with a VLC player plugin. Sound was played back at 74 dB (Fast, A, re 20 μPa, Voltcraft SL-451, Conrad, Hirschau, Germany) at 30 cm from a loudspeaker (Blaupunkt, CB4500, Hildesheim, Germany) suspended from the ceiling at 50 cm above the cage (directly above the video monitor, see Fig. 3). We had decided on this position, because positioning the loudspeaker behind the monitor would have negatively affected the sound quality. Visual stimulation can attract the perceived location of spatially discordant but temporally synchronous auditory stimulation (Chen and Vroomen 2013). This phenomenon, known as spatial ventriloquism, has been demonstrated in species as diverse as humans, frogs, spiders and birds (Narins et al. 2005; Lombardo et al. 2008; Chen and Vroomen 2013; Kozak and Uetz 2016). Little is known about cross-modal integration in zebra finches, but in another bird species, spatial ventriloquism was found to take place over a distance of one meter between the auditory and visual stimulus (Lombardo et al. 2008). The loudspeaker above the cage of the audio-only condition was connected to the computer of the audio-pixel condition. Each cage was placed on a table in a sound-attenuated room (125 × 300 × 240 cm). A webcam (Renkforce RF-4805778, Conrad, Hirschau, Germany) was installed next to the cage to record the tutees’ behaviour in the cage.

### Song recordings tutees

All tutees were recorded once as juveniles at 65 dph (*X* ± SE: 64.6 ± 0.9) and once as young adults after 100 dph (*X* ± SE: 116 ± 12). For the first recording at 65 days post-hatching, male and female tutees were jointly moved into a cage (76 × 45 × 45 cm) in a sound-attenuated recording room (125 × 300 × 240 cm) between 12:00 and 13:00. A Sennheiser MKH40 microphone (Wedemark, Germany), connected to a TASCAM DR-100MKiii recorder, was hanging at 50 cm above the perch in the recording cage. Recordings were made with a 96 kHz sampling frequency. Recordings were made continuously during the next morning, after which birds were moved back to the experimental set-up. After 100 days post-hatching, male tutees were recorded again using the same recording set-up and the same procedure, but now males were housed singly in the recording room. There were 42 birds that produced more than 20 songs during this recording session. Only song of these birds was used in the song analysis (one tutee from the audio–video and one tutee from the audio-pixel treatment did not sing enough).

### Song analysis

An overview of all song analysis measures can be found in Table 2. In almost all tutees, the song that was recorded at 65 days post hatching was still too variable to recognize syllables and motifs. All analyses were therefore conducted on the song recordings made after 100 dph.

**Table 2:** Overview of song analysis parameters used in this study and the sample that was used to calculate them

Parameter	Definition	Sample per bird used to calculate the parameter
Typical motif	Most frequently produced motif	20 random songs
Full motif	Motif with highest # different syllables in bird’s repertoire	20 random songs
Total number of syllables	# syllables in a tutee’s typical motif	Typical motif
Number of unique syllables	# unique syllables in a tutee’s full motif	Full motif
Linearity	$\frac{\#\mathrm{ different \, syllables}/\mathrm{song}}{\#\mathrm{transition\, types}/\mathrm{song}}$	20 random songs
Consistency	$\frac{\mathrm{total }\#\mathrm{ typical\, transitions}}{\mathrm{total }\#\mathrm{ of\, transitions}}$	20 random songs
Human observer similarity score model–tutee	$\frac{\Sigma\, \mathrm{ similarity \, scores \, for \, all \, tutee \, syllables}}{\#\mathrm{ tutee \, syllables}*3 \,(\mathrm{max. score})*\#\mathrm{ observers}}$	Full motif
Human observer similarity score tutee–model	$\frac{\Sigma\,\mathrm{ similarity \, scores \, for \, all \, tutee \, syllables}}{\#\mathrm{ tutee \, syllables}*3\, (\mathrm{max. score})*\#\mathrm{ observers}}$	Full motif
SAP similarity score tutor–tutee	SAP similarity scores comparing tutors’ to tutees’ motifs	10 random motifs
SAP similarity score tutee–tutor	SAP similarity scores comparing tutees’ to tutors’ motifs	10 random motifs
Luscinia similarity score	1 − Luscinia distance score for comparison of tutor and tutee motifs	10 random motifs
SAP stereotypy score	SAP similarity scores for the comparison between tutee motifs	10 random motifs
Luscinia stereotypy score	1 − Luscinia distance scores for the comparison between tutee motifs	10 random motifs

#### Song and motif selection

**Fig. 2:**
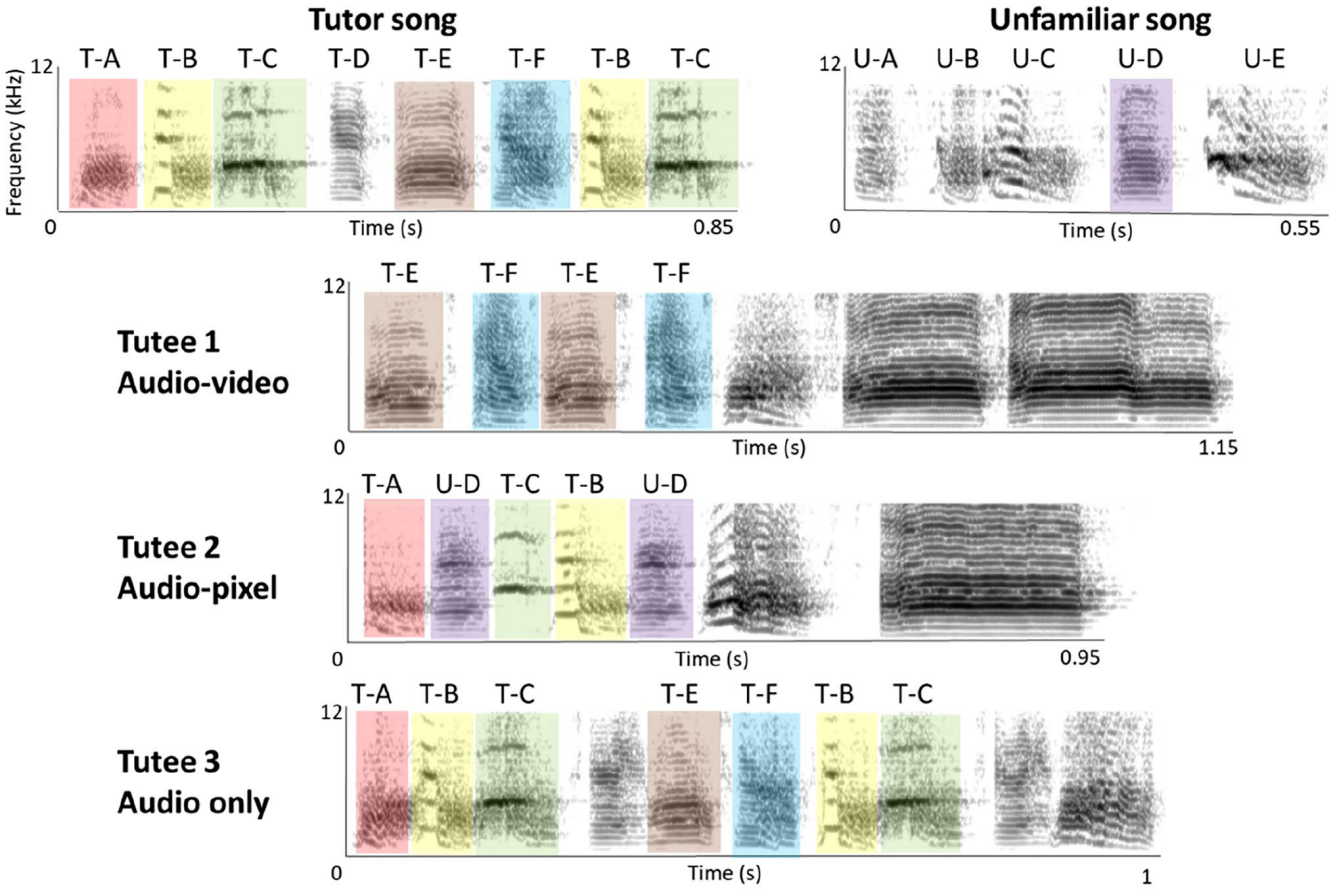
Spectrograms of the full motif of the tutor, the unfamiliar full motif of another adult male and three tutees from one tutor group. Letters above tutor and unfamiliar song spectrograms indicate how syllables were labelled with letters for further analyses. Human observer similarity between tutor/unfamiliar song and tutees was scored on a scale from 0 to 3. Syllables marked with the same colour and with the same label above them had a total similarity score of 4 or higher when the similarity scores of all three observers for this comparison were summed up

For all sound analyses and sound editing, we used spectrograms calculated with the Praat-software (fast Fourier transformations with 1000 time and 250 frequency steps, 0.005 s window length, dynamic range 55 dB, Gaussian window, Praat v. 6.0.19, Boersma and Weenink 2018). First, all songs were cut out of the recording sessions’ audio files saving all songs per male into one folder to then randomly select 20 songs from this folder (with custom-written software by Niklas J. Tralles). As mentioned above, a song was defined as one or several motifs separated from other sounds by more than 2 s of silence or when a motif was starting with additional introductory notes. This sample was used to calculate linearity and consistency, and to identify a tutee’s ‘typical’ and ‘full’ motif (a motif was defined as the repeated syllable sequence in a song). The typical motif was defined as the motif encountered most often in the 20 randomly selected songs and the full motif as the motif with the highest number of different syllables. The full motifs were used for the human observer similarity scoring and to determine the total number of syllables in the tutee’s repertoire (see below). For each tutee, we labelled different syllables with different letters (see Fig. 2). From the 20 songs, we selected a new smaller subsample consisting of 10 out of the 20 randomly selected songs (again using the custom-written software making a random selection from each folder). A random number generator (http://www.random.org) was then used to randomly select one motif from each of these ten songs. Using Praat-software, these ten motifs were cut out of the recordings, filtered with a band stop filter from 0 to 420 Hz, and the amplitude was normalized using the ‘scale peak’ function. Introductory notes that did not occur with every repetition of the motif were not considered to be part of the motif and cut off before proceeding further with the analyses. These ten motifs were used for the SAP and Luscinia similarity and stereotypy scores (see below).

#### Song structure and performance

For each tutee, we determined the total number of syllables in the typical motif and the number of unique syllables in the full motif by visually inspecting the spectrograms in Praat (settings as described above). We calculated sequence linearity and sequence consistency (Scharff and Nottebohm 1991) for the 20 randomly selected songs. Sequence linearity was calculated by dividing the number of different syllables (e.g. A, B, C …) by the number of different transitions between syllables (e.g. AB, AC, BC …) in a song. This measure indicates how stereotyped syllables are ordered in a song, with more stereotyped songs yielding higher scores. Consistency was determined by first noting all transitions in the 20 songs. For each syllable, the typical transition was then determined by looking at the most frequently encountered transition from this syllable. The total number of occurrences of typical transitions was then divided by the total number of transitions encountered in the 20 randomly selected songs. Again, more stereotyped songs receive a higher score.

#### Similarity between tutee and tutor song

For zebra finch song, the literature up until 1999, including the studies most relevant to this study (Bolhuis et al. 1999; Houx and ten Cate 1999b), mostly used visual inspection of spectrograms by human observers to assess song similarity between tutors and tutees. This is why we also decided to assess song similarity using human observers. Since 2000, automated digital measurement methods, such as Sound Analysis Pro (SAP, Tchernichovski et al. 2000, specifically developed to assess zebra finch song learning) and Luscinia (Lachlan et al. 2010) have regularly been used. An often-mentioned advantage of automated song similarity assessment is that it compares song objectively. However, human observer similarity scoring is also objective when using observers that are blinded to the origin and/or expected outcome of the spectrogram comparisons, which was the case in this study. Moreover, both of the aforementioned automated comparison methods were validated against comparisons done by human observers (Luscinia: Lachlan et al. 2010; SAP: Tchernichovski et al. 2000), which the developer of SAP considers preferred over automated methods (Tchernichovski 2011). In this study, we will therefore primarily use similarity scoring by human observers to assess song learning success in the birds from the different treatments. However, to allow cross-study comparisons, we also assessed song similarity using Luscinia and Sound Analysis Pro (for details see below). We calculated the correlation between the similarity scores obtained with the three different methods to find out whether they provide a similar outcome.

For the human ratings of similarity, we followed the methods used by Houx and ten Cate (1999a), but compared motifs at the syllable level (continuous sounds separated by at least 5 ms of silence), while Houx and ten Cate (1999a) compared motifs at the element level (sounds separated from other sounds by either an observable gap of silence on the spectrogram or by an abrupt change in frequency or structure, meaning that one syllable can consist of several elements). Based on previous studies, we expected poor song copying in the audio tutees (Price 1979; Eales 1989) and depending on whether videos would or would not sufficiently substitute for live tutors potentially in the other treatment groups too. In poorly copied and isolate-like song, determining element boundaries can be difficult, for instance due to a higher variance in frequency patterns than in normal song (Price 1979) while determining syllable boundaries is more straightforward. For this reason, we decided to assess similarity on the syllable level. For visual scoring, a PowerPoint presentation was created where each slide contained two spectrograms: on top the full motif of the tutee (labelled ‘tutee’) and below a second spectrogram labelled ‘model’. The model song was either from the tutor or from the tutor of another tutor group (unfamiliar to the tutee). Each tutee was thus compared with two models: the actual tutor and an unfamiliar control model (the tutor of another group). We included the unfamiliar song to analyse the level of syllable sharing between two birds from the same colony that occurs by chance. Syllables were labelled with different letters by one of us (JV) and these letters were placed below each of the two spectrograms on each slide. Three independent observers (Ph.D. candidates at the Leiden lab not involved in this study and with varying experience in working with spectrograms of zebra finch song) received the PowerPoint presentation. For each syllable in the tutee’s repertoire, the observers were asked to indicate which syllable of the model it resembled most by paying attention to frequency pattern, duration, shape and position with respect to neighbouring syllables, and to then indicate the degree of similarity on a four-step scale (0 = ‘no similarity at all’, 1 = ‘slight similarity’, 2 = ‘moderate similarity’ and 3 = ‘very strong similarity’). Observers were given no information on tutees’ treatment groups and whether a model song was from the tutor or from another male. To assess inter-observer reliability, we first normalized the scores per observer (for each score we subtracted the mean of all scores of this observer and then divided it by the standard deviation of the scores of the observer). We calculated repeatability using a one-way ANOVA (following Lessells and Boag 1987) with the similarity score as the dependent variable and tutee ID as factor. The repeatability estimate *r* of the normalized scores was moderate (tutor–tutee: *r* ± SE = 0.54 ± 0.09, *F*_2_,_39_ = 4.45, *p* < 0.01; tutee–tutor: *r* ± SE = 0.50 ± 0.09, *F*_2_,_39_ = 4.03, *p* < 0.01). The difference between observers mainly had to do with how strict observers were regarding poorly copied syllables. To capture this best and to have one value for further analyses that would integrate all observer values, we decided to work with the total sums of similarity scores (of all three observers) for a tutee divided by the potential maximum score a bird could receive from three observers (the sum of the similarity scores of all three observers for all pairwise syllable comparisons of a particular model–tutee comparison). This score thus corrected for between individual differences in syllable numbers, thereby providing a measure combining the proportion of syllables copied as well as a weighing of their similarity.

Syllable sharing and similarity values are affected by the direction of such a comparison if model and tutee differ in total number of syllables and therefore can be assessed in two ways (1) the proportion and similarity of the model’s syllables copied by the tutee (“similarity score model–tutee”) and (2) the proportion and similarity of the tutee’s syllables shared with the model (“similarity score tutee–model”). The tutee–model comparison was included as tutees can differ in how many syllables they improvise in addition to song copied from a tutor (Williams 1990). To clarify, a tutee that has accurately copied the syllables ABC from a tutor with the song ABCDE would get a higher score for the tutee–model comparison than for the model–tutee comparison. A tutee that sings ABCDEFG (with ABCDE accurately copied from the tutor and F and G improvised) would get a higher score for the model–tutee comparison than for the tutee–model comparison. For the model–tutee comparison, for each model syllable, the ID and similarity score of the tutee syllable that received the highest score was noted, and these scores were summed. If two or more tutee syllables received the same similarity score, we noted this score once, but the scores for all tutee syllables were included in the tutee–model comparison. For each motif, the scores of all three observers were then summed up and divided by the maximum possible score (see Table 2 for full formula).

For the automatic, quantitative song comparisons, we compared each of 10 randomly selected motifs of a tutee to each of 10 randomly selected motifs of its tutor using both Luscinia (version 2.16.10.29.01) and Sound Analysis Pro (MxN comparison, default settings tuned for zebra finch, per tutor–tutee pair amplitude thresholds were adjusted for correct syllable segmentation, version 2011.104). A difference between the two methods is that SAP uses a linear time-warping algorithm to align two signals for comparison, while Luscinia uses dynamic time-warping (DTW) which searches for the optimal alignment of two time series irrespective of how warped they have been in time (Lachlan et al. 2010). Similarity assessment in Sound Analysis Pro is based on five acoustic features: pitch, frequency modulation, amplitude modulation, goodness of pitch and Wiener entropy. Like with the human observer similarity scores, SAP similarity scores are influenced by the direction of the comparison. For each possible comparison, we calculated the asymmetric similarity score for the tutor to tutee comparison (SAP similarity score tutor–tutee), which indicates the percent of sounds in the tutor’s song that are observed in the tutee’s song, as well as for the tutee to tutor comparison (SAP similarity score tutee–tutor), which indicates the percent of sounds in the tutee’s song that are observed in the tutor’s song. We used the median value of these scores as the quantitative measure of similarity (henceforth ‘SAP similarity score’), as our sample size of birds was too small to create a good-fitting model for the similarity scores of all comparisons and as the SAP scores were not normally distributed and bound between 0 and 100. Luscinia also calculates global similarity but works with a dynamic time-warping algorithm to calculate acoustic distance scores between tutee–model pairs. We chose the acoustic features ‘mean frequency’, ‘fundamental frequency’ and ‘fundamental frequency change’ for the acoustic distance calculations (following Lachlan et al. 2016). We also included ‘time’ in the analysis, which allows for flexible comparison of signals that vary in length. The output of the DTW analysis is a distance measure between 0 and 1 for all possible pairs of motifs. Unlike the human observer and SAP similarity scores, this is a symmetric score, so there is no difference between a model to tutee or tutee to model comparison. We used the median distance score for each tutee–model pair, and transformed it into a similarity score by calculating 1-distance score (henceforth ‘Luscinia similarity score’), so that, like with the other scores, a higher score indicates a higher similarity. As a measure of song stereotypy and to get an indication of how similar the 10 randomly selected tutee motifs were to each other, we also compared the 10 tutee motifs to each other in Sound Analysis Pro and Luscinia. We used the same settings for this comparison as for the tutor to tutee comparisons. In Sound Analysis Pro, we calculated the median of the symmetric similarity score for the comparison of the 10 tutee motifs. This will be referred to as the ‘SAP stereotypy score’. In Luscinia, we used the median distance score for the comparison of the 10 tutee motifs and then calculated 1 − this distance score, again so that a higher score indicates a higher similarity. This score will be referred to as the ‘Luscinia stereotypy score’.

### Behaviour recording and analysis

**Fig. 3:**
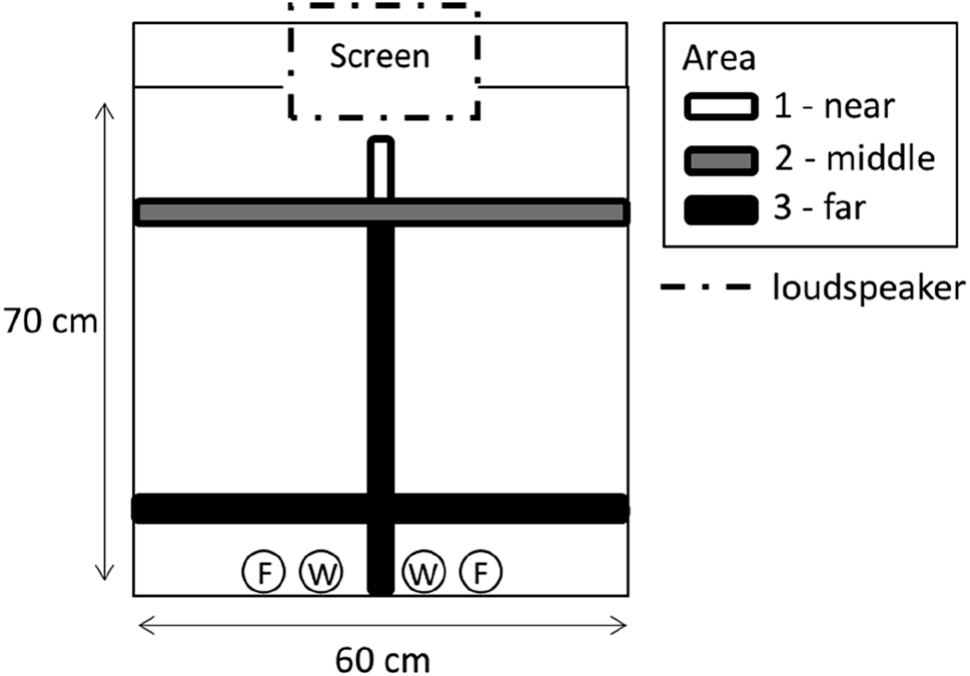
Schematic top view of the experimental set-up. In the set-up for the audio group, there was no screen next to the cage. For the behaviour observations, we divided the cage into three areas, with 1 being the perch area nearest to the screen (8 cm of perch), 2 being an intermediate area (60 cm of perch) and 3 the perch area furthest from the screen (104 cm of perch). The dotted rectangle indicates the location of the loudspeaker (hanging 50 cm above the cage). F = food, W = water. Food and water bottles were placed on the floor of the cage

For the 30 days of tutoring, daily web-cam (Renkforce RF-4805778, Conrad, Hirschau, Germany) recordings were made of the tutoring sessions at 8:15, 12:15 and 16:15. For six tutor groups (18 male–female tutee dyads) that were tutored with tutoring schedule 2 (see Table 1), videos from every 5th day were coded using BORIS software (version 7.5.1). Coding was done by two of us (IvH and RJ) that first scored the same video’s independently until they reached an inter-observer reliability value of *K* > 0.9 (Cohen’s Kappa calculated by BORIS). After this, they each coded different videos (N.B. for these videos observer blinding was not possible, as filming and scoring the approach towards the stimuli showed the stimuli. However, observer biases are playing out strongest with ambiguous or continuous categories, but less so for discrete units such as these spatially separated perches). The observers scored the position of the tutees in the different areas of the cage during stimulus presentation (see Fig. 3). This was used to calculate the proportion of the observed time that tutees spent in the different areas corrected for perch length in each area ((time spent_area *x*/_length perch_area *x*_)/(total time/total cm perch length)). In addition, we also scored the amount of times the birds left the perches to fly directly up and against the screen. For the audio condition, the amount of times the tutees flew up and against the location of the screen was scored, even though the audio birds did not have a screen next to their cage.

### Statistical analysis

RStudio (R: version 3.5.1) was used for all statistical analyses. To assess tutee engagement with the stimuli, the proportion of time spent in different cage areas (corrected for perch length in that area) was arcsine square root transformed before analyses to meet model assumptions. We then created linear mixed models (LMMs, package *lme4*: Bates et al. 2014) and started with a null model that only included ‘TutorGroup’ (Number of the tutor group) as a random factor. We then added fixed effects in the following order: ‘area’ (1, 2 or 3), ‘treatment’ (audio–video, audio-pixel or audio), the interaction between ‘area’ and ‘treatment’, and ‘sex’ (sex of the tutee: male or female). We used ANOVA’s to check whether each of these fixed effects led to a significant improvement of the model. For the number of screen approaches, we created negative binomial generalized linear mixed models (GLMMs). We started with a null model with only ‘TutorGroup’ as random factor, then added fixed effects in the following order: ‘Treatment’, ‘Sex’ and ‘Tutoring day (number of days since the tutee was moved to the experimental set-up)’ and used an ANOVA to test whether these factors significantly improved the model.

For the stereotypy and human observer, SAP and Luscinia similarity scores, we built linear mixed-effects models (LMMs). Human observer, SAP and Luscinia scores were arcsine square root transformed before analyses to meet model assumptions. To calculate the correlation between the three different similarity scores (human observers, SAP and Luscinia), we calculated the Pearson correlation coefficient after a square root transformation of the human observer scores to meet assumptions of normality. Generalized linear mixed-effect models (GLMMs) with a Poisson distribution and log-link function were created for the total number of (unique) syllables. For the analysis of all song parameters, we started with a null model with only ‘TutorGroup’ (ID of the tutor group) as a random factor. We then added ‘Schedule’ (the three different tutoring schedules) and ‘Treatment’ as fixed effects. We used ANOVA’s to test whether adding each of these model terms led to a significant improvement compared to the simpler model. As the human observer similarity scores were our main parameter of interest for assessing song learning success and we were interested in the similarity scores attained by the tutees from the different tutoring treatment groups, we still ran a model with ‘Treatment’ as fixed factor for the human observer similarity scores even if this did not significantly improve the model. To test whether tutees had a higher score for human observer similarity with the song of the tutor than with the unfamiliar song of another male, we built LMMs and tested whether adding ‘model’ (tutor or unfamiliar)’ as fixed factor significantly improved the null models (with ‘TutorGroup’ and ‘Bird ID’ as random factors).

For all models, a Shapiro–Wilk test was used to test whether the models’ residuals followed a normal distribution. Post-hoc tests with Tukey adjustment for multiple comparisons were performed for between treatment comparisons (package *emmeans*: Lenth et al. 2018).

### Ethics statement

Following European and national law, all procedures were reviewed and approved by the Leiden University Committee for animal experimentation, Leiden University Animal Welfare Body and the Centrale Commissie voor Dierproeven (CCD) of the Netherlands (Permit number AVD1060020186606).

## Results

### Tutee behaviour

**Fig. 4:**
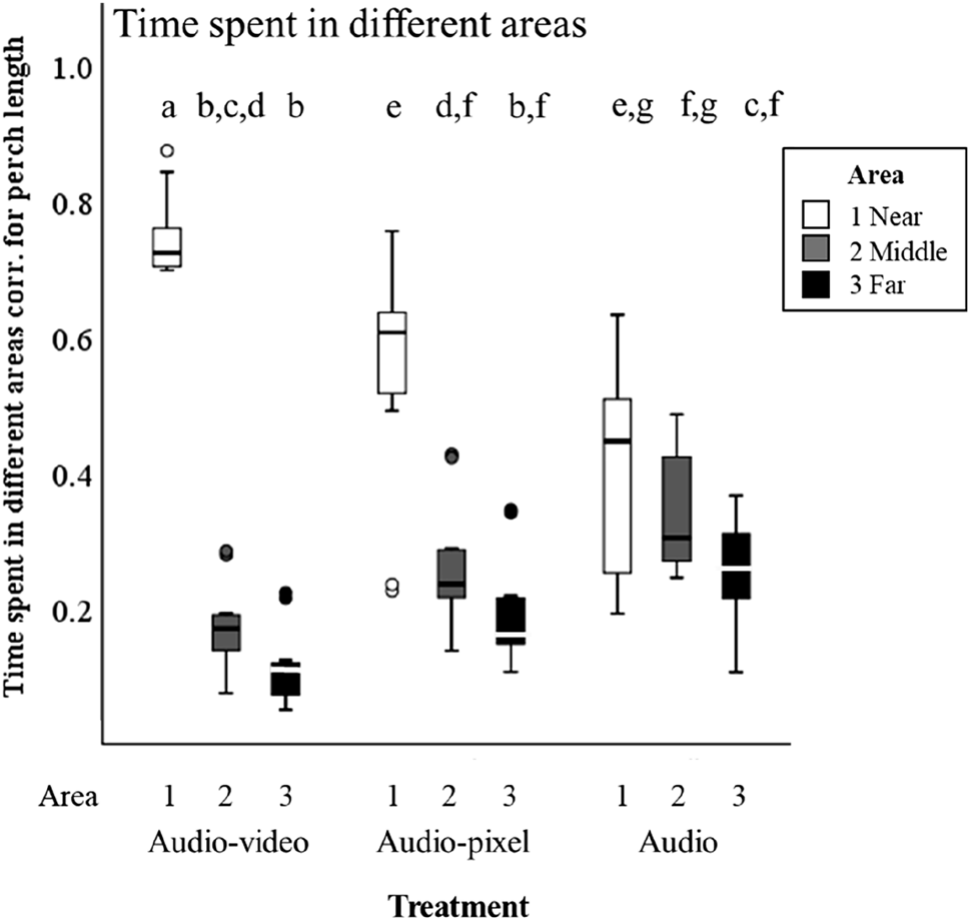
Proportion of time spent in the different cage areas, corrected for the total perch length in that area. Box plots indicate the median (mid-line), interquartile range (box), and 1.5 times the interquartile range (whiskers). Data points beyond this range are plotted as individual points. Different letters above boxes indicate a significant difference of *p* < 0.05 according to post hoc tests (see Appendix, Table 11), LMM see Table 3

**Fig. 5:**
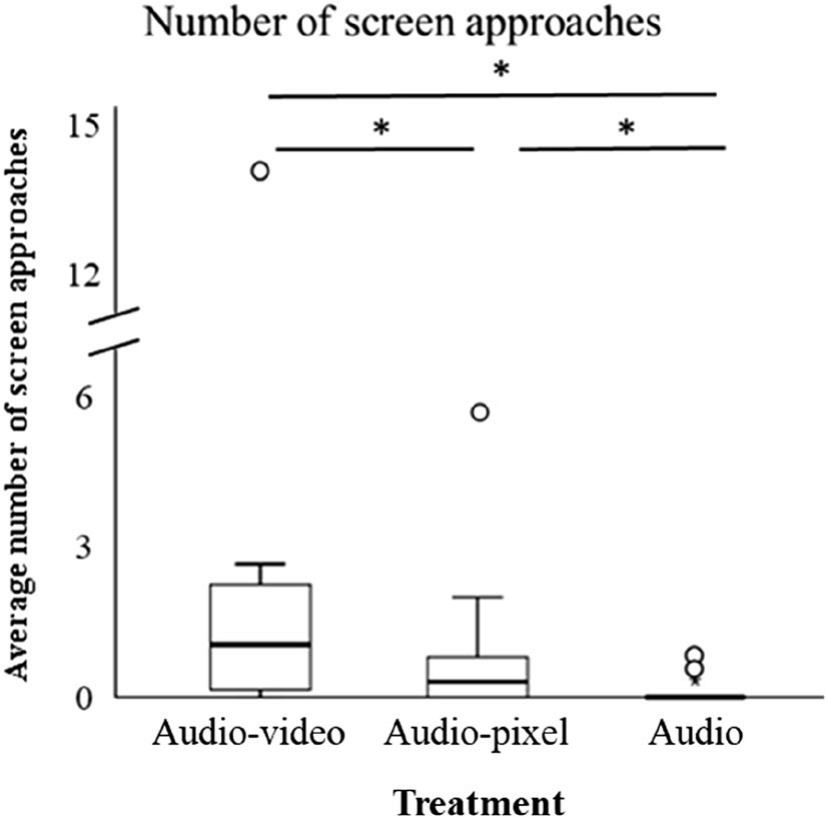
The average number of direct screen approaches during the stimulus presentations (values are the average per tutee for the three scored presentations per recording day (every fifth day of the tutoring period three (out of four) tutoring sessions were recorded and scored)). *Indicates *p* < 0.05, GLMM see Table 4

During the tutoring sessions, birds did not use all areas in the cage equally often (Fig. 5). Birds in all groups showed a bias towards area 1 which was closest to where the stimuli could be seen and heard. To test whether this engagement with the stimuli differed across treatments, we analysed the proportion of time during the tutoring sessions that the tutees spent in the different areas of the cage corrected for the perch length in that area. The proportion of time spent was affected by area, treatment and the interaction between area and treatment: tutees spent a significantly higher proportion of time in area 1 (near) in the audio–video group than in the audio-pixel and audio group. Besides, in the audio–video and audio-pixel group, more time was spent in area 1 (near) than in area 2 (middle), while this difference was not found in the audio group (best model included ‘treatment’, ‘area’ and the interaction between ‘treatment’ and ‘area’, see Table 3 and Fig. 4).

**Table 3:** Details of best model (LMM) for the proportion of time spent in different areas of the cage, corrected for the perch length in that area

Response variable^a^	Model term	Level	Estimate	SE	*t*
Prop. of time spent	Intercept		0.69	0.03	20.49
corrected for perch	Treatment				
length		*Audio–video*	0.32	0.05	6.72
		*Audio-pixel*	0.14	0.05	2.95
	Location				
		*Area 2 (middle)*	− 0.07	0.05	− 1.56
		*Area 3 (far)*	− 0.17	0.05	− 3.61
	Location × treatment				
		*Area 2* × *Audio–video*	− 0.51	0.07	− 7.61
		*Area 3* × *Audio–video*	− 0.50	0.07	− 7.48
		*Area 2* × *Audio-pixel*	− 0.23	0.07	− 3.34
		*Area 3* × *Audio-pixel*	− 0.21	0.07	− 3.16

^a^LMM with random factor ‘Tutor group’. For post-hoc comparisons see Appendix, Table 11

The amount of times that the tutees flew up to the screen (or the location of the screen in the audio group) differed between the treatment groups: there were more direct screen approaches in the audio–video condition than in the audio-pixel and audio condition, and more screen approaches in the audio-pixel than in the audio condition (model including ‘treatment’ significantly better than model without treatment, *N* = 36, *χ*^2^ = 40.62, *p* < 0.01, see Table 4 (also for post-hoc test results) and Fig. 5). The number of direct screen approaches did not differ between the male and female tutee (adding ‘sex’ did not significantly improve the model, *N* = 36, *χ*^2^ = 0.73, *p* = 0.39) and did not change over time (adding ‘Tutoring day’ also did not significantly improve the model, *N* = 36, *χ*^2^ = 0.12, *p* = 0.73).

**Table 4:** Details of best model (GLMM) for the amount of screen approaches

Response variable^a^	Model term	Level	Estimate	SE	*z*	*p*
Number of screen	Intercept		− 4.15	0.70	− 5.89	**< 0.01**
approaches	Treatment					
		*Audio–video*	3.46	0.66	5.21	**< 0.01**
		*Audio-pixel*	2.45	0.70	3.51	**< 0.01**

Significant *p*-values are given in bold

^a^Negative binomial GLMM with random factor ‘Tutor group’. Significant post-hoc comparisons: audio vs. audio–video: estimate: − 3.46, SE: 0.66, *z*: − 5.21, *p* < 0.01, audio vs. audio-pixel: estimate: − 2.45, SE: 0.70, *z*: − 3.51, *p* < 0.01, audio–video vs. audio-pixel: estimate: 1.02, SE: 0.38, *z*: 2.67, *p* < 0.05

### Song structure and performance

The song structure and performance parameters (total number of syllables, number of unique syllables, linearity and consistency) did not differ between the treatment groups (models including ‘treatment’ were not significantly better than null models, see Table 5). Presentation schedule affected none of the parameters but linearity, which differed between the three tutoring schedules and was higher in schedule 1 (fewer presentations) than in the other schedules (see Table 6C, model including ‘schedule’ significantly better than null model, *N* = 42, *χ*^2^ = 8.80, *p* = 0.01, best models for each parameter in Table 6).

**Table 5:** Mean values of song structure and performance parameters and details on ANOVA for comparison between null model and model including ‘treatment’ as a fixed effect

	Tutor (not in models)	Audio–video	Audio-pixel	Audio	ANOVA null model and model with ‘treatment’		
	Mean ± SD	Mean ± SD	Mean ± SD	Mean ± SD	*N*	*χ*^2^	*p*
Total nr syllables	6.33 ± 1.44	5.08 ± 1.38	6.46 ± 1.76	5.25 ± 2.34	42	2.56	0.28
Nr unique syllables	5.25 ± 1.60	4.60 ± 1.30	4.93 ± 1.44	4.42 ± 0.51	42	0.40	0.82
Linearity	0.46 ± 0.12	0.41 ± 0.11	0.40 ± 0.10	0.44 ± 0.09	42	0.85	0.66
Consistency	0.94 ± 0.04	0.89 ± 0.08	0.90 ± 0.07	0.92 ± 0.08	42	0.77	0.68

In the models, only the data from the tutees from the different tutoring treatments was compared (the tutor data was not included in the models)

**Table 6:** Details of best models for the song structure and performance parameters

Response variable	Model term	Level	Estimate	SE	*z*
(A) Total number of syllables^a^	Intercept		1.72	0.07	25.16
(B) Number of unique syllables^a^	Intercept		1.54	0.07	21.57
(C) Linearity^b^	Intercept		0.51	0.04	14.49
	Schedule				
		*Schedule 2*	− 0.12	0.04	− 3.04
		*Schedule 3*	− 0.12	0.05	− 2.47
(D) Consistency^b^	Intercept		0.90	0.02	49.34

^a^GLMM with a Poisson distribution and random factor ‘Tutor group’

^b^LMM with random factor ‘Tutor group’

### Similarity to tutor song

#### Comparison different similarity assessment methods

There was a significant correlation between the human observer and the Luscinia similarity score, but not between the human observer and the SAP similarity score or the SAP and the Luscinia similarity score (see Table 7), suggesting that these measures pick up on different dimensions of song similarity. It is important to note, however, that the correlation between the human observer similarity scores on the one hand, and the SAP and Luscinia scores on the other hand is influenced by the different samples that were used to calculate these scores (1 typical motif for the human observer scores and 10 randomly selected motifs per tutee for the SAP and Luscinia scores). In subsequent paragraphs, we will present the results of all three methods, although, as mentioned before, we will primarily focus on the results from the human observer similarity scoring to determine whether song learning success was affected by the different tutoring treatments.

**Table 7:** Pearson correlation coefficients for the human observer similarity scores (square-root transformed to meet assumptions of normality), the median SAP similarity scores and the median Luscinia similarity scores for the tutor to tutee comparison

Comparison	*N*	*r*	*p*
Human observer sim. score—SAP sim. score	42	0.04	0.98
Human observer sim. score—Luscinia sim. score	42	**0.57**	**< 0.01**
SAP sim. score—Luscinia sim. score	42	0.14	0.44

Significant values are given in bold

#### Similarity scores for the comparison between tutor and tutee songs

To find out whether the tutees had learned from the tutor, we checked whether their song was more similar to the tutor song than to an unfamiliar song. The human observer similarity scores for the tutor to tutee and tutee to tutor comparisons were significantly higher than the similarity scores for the comparisons with an unfamiliar song (model with ‘model (tutor or unfamiliar)’ was significantly better than null model, model to tutee comparison: *N* = 42, *χ*^2^ = 5.39, *p* = 0.02, Table 8A, tutee to model comparison: *N* = 42, *χ*^2^ = 4.75, *p* = 0.03, Table 8B). As this means that tutees’ songs were more similar to their tutor’s song than would be expected by random sharing in the colony, we assume that the tutees learned at least some aspects from their tutors. For all subsequent analyses, we proceed with comparisons between tutor and tutees only.

**Table 8:** Details of best models for the arcsine square-root transformed human observer similarity scores for the comparison of the model songs to the tutee songs (A) and the tutee songs to the model songs (B)

Human observer similarity scores					
Response variable	Model term	Level	Estimate	SE	*t*
(A) Model–tutee^a^	Intercept		0.52	0.02	21.63
	Model				
		*Unfamiliar*	− 0.08	0.03	− 2.34
(B) Tutee–model^a^	Intercept		0.57	0.02	23.41
	Model				
		*Unfamiliar*	− 0.08	0.03	− 2.18

^a^LMM with random factors ‘Tutor group’ and ‘Bird ID’

**Fig. 6:**
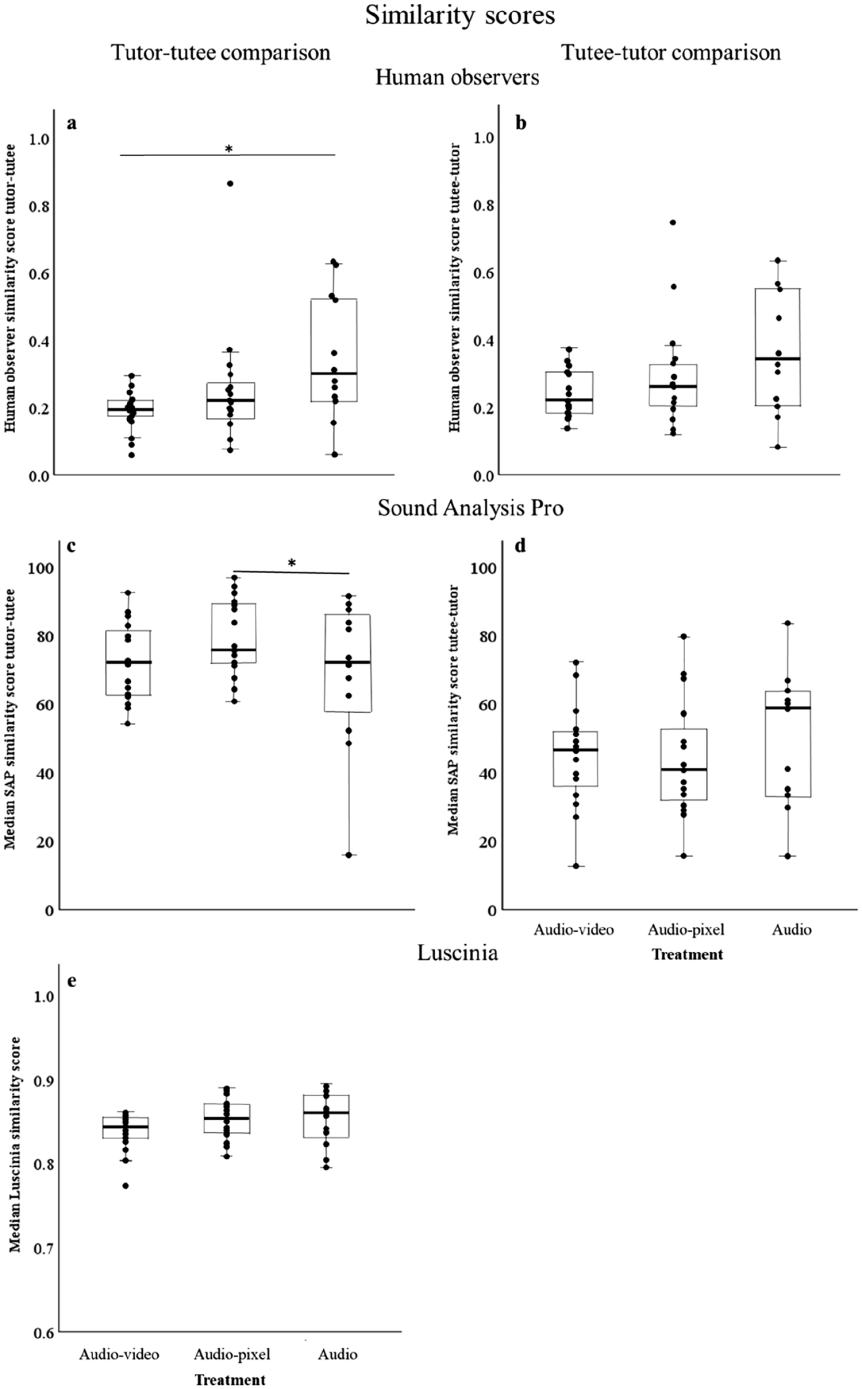
Graph showing the human observer similarity score for the tutor–tutee (**a**) and the tutee–tutor comparison (**b**), the SAP similarity score for the tutor–tutee (**c**) and the tutee–tutor (**d**) comparison and the Luscinia similarity score for the symmetric tutee and tutor comparison (e). *Indicates *p* < 0.05, LMMs see Table 9. NB human observer and SAP similarity scores calculate how much of one signal can be found in another signal. Therefore, when comparing two signals, two different comparisons can be made [what proportion of the tutor motif is found in the tutee motif (tutor–tutee) and what proportion of the tutee motif is found in the tutor motif (tutee–tutor)]. Luscinia does not calculate how much of one signal can be found in another signal, but calculates how dissimilar two signals are

In the comparison of the syllables in the tutor’s repertoire to those in the tutee’s repertoire (tutor–tutee comparison), the human observer similarity scores differed between the treatment groups: these scores were higher in the audio group than in the audio–video group (model including ‘treatment’ was significantly better than null model, *N* = 42, *χ*^2^ = 6.60, *p* = 0.04, see Table 9A (also for post-hoc test results) and Fig. 6). The tutor–tutee similarity scores did not differ between the tutoring schedules (model including ‘schedule’ was not significantly better than null model, *N* = 42, *χ*^2^ = 3.34, *p* = 0.19). In the comparison of the syllables in the tutee’s repertoire to those in the tutor’s repertoire (tutee–tutor comparison), human observer similarity scores were also highest in the audio group (see Table 9A), but these similarity scores were not significantly affected by the different tutoring treatments [adding ‘treatment’ as fixed factor did not significantly improve the null model (*N* = 42, *χ*^2^ = 4.72, *p* = 0.09)]. The tutee–tutor similarity scores also did not differ between the tutoring schedules [adding ‘schedule’ did not significantly improve the null model (*N* = 42, *χ*^2^ = 2.27, *p* = 0.32)].

**Table 9:** Details of models with ‘Treatment’ as fixed factor for the arcsine square root transformed human observer similarity scores (A) and the best models for the arcsine square root transformed SAP (B) and Luscinia (C) similarity scores

Response variable	Model term	Level	Tutor–tutee			Tutee–tutor		
			Estim	SE	*t*	Estim	SE	*t*
(A) Human observers sim. scores^a^	Intercept		0.62	0.05	12.18	0.64	0.05	14.05
	Treatment							
		*Audio–video*	− 0.17	0.07	− 2.58	− 0.13	0.06	− 2.16
		*Audio-pixel*	− 0.10	0.07	− 1.48	− 0.07	0.06	− 1.18
(B) SAP sim. scores^b^	Intercept		1.00	0.05	18.59	1.07	0.04	27.07
	Treatment							
		*Audio–video*	0.06	0.05	1.01			
		*Audio-pixel*	0.16	0.05	3.01			
(C) Luscinia sim. scores^c^	Intercept		1.19	0.01	109.69			
	Treatment							
		*Audio–video*	− 0.024	0.01	− 2.15			
		*Audio-pixel*	− 0.001	0.01	− 0.07			

^a^LMMs with random factor ‘Tutor group’. Significant post-hoc comparison tutor–tutee: audio vs. audio–video: estimate: 0.17, SE: 0.07, *t*: 2.56, *p* = 0.04

^b^LMMs with random factor ‘Tutor group’. Significant post-hoc comparison tutor–tutee: audio vs. audio-pixel: estimate: − 0.16, SE: 0.06, *t*: − 2.99, *p* = 0.02. For the tutee–tutor comparison, ‘treatment’ was not included in the best model

^c^LMMs with random factor ‘Tutor group’

The SAP similarity scores for the comparison of the tutor song to the tutee song (SAP similarity scores tutor–tutee) differed between the treatment groups and did not differ between the tutoring schedules: the tutor–tutee similarity scores were higher in the audio-pixel group than in the Audio group (model with ‘schedule’ was not significantly better than null model: *N* = 42, *χ*^2^ = 2.89, *p* = 0.24, while model with ‘treatment’ was significantly better than null model: *N* = 42, *χ*^2^ = 8.73, *p* = 0.01, see Table 9B (also for post-hoc test results) and Fig. 6C). For the comparison of the tutee’s songs with their tutor’s song, the Sound Analysis Pro similarity scores (SAP similarity score tutee–tutor) did not differ between the tutoring schedules or the tutoring treatments (model with ‘schedule’ was not significantly better than null model: *N* = 42, *χ*^2^ = 0.38, *p* = 0.83, model with ‘treatment’ was not significantly better than null model: *N* = 42, *χ*^2^ = 1.12, *p* = 0.57, see Table 9B for best model).

The different treatment conditions affected the Luscinia similarity scores, but the post-hoc test did not detect any significant differences between two treatment groups (model including ‘treatment’ was significantly better than the null model, *N* = 42, *χ*^2^ = 6.46, *p* = 0.04, see Table 9C and Fig. 6). Luscinia similarity scores were not affected by the different tutoring schedules (model including ‘schedule’ was not significantly better than the null model, *N* = 42, *χ*^2^ = 0.89, *p* = 0.64).

Overall, the similarity between tutor and tutee song was highest for the audio tutees for all methods and comparisons, except for the SAP similarity scores for the tutor–tutee comparison (see Table 9 and Fig. 6). For this comparison, similarity scores were highest in the Audio-pixel group.

#### SAP and Luscinia stereotypy scores

**Fig. 7:**
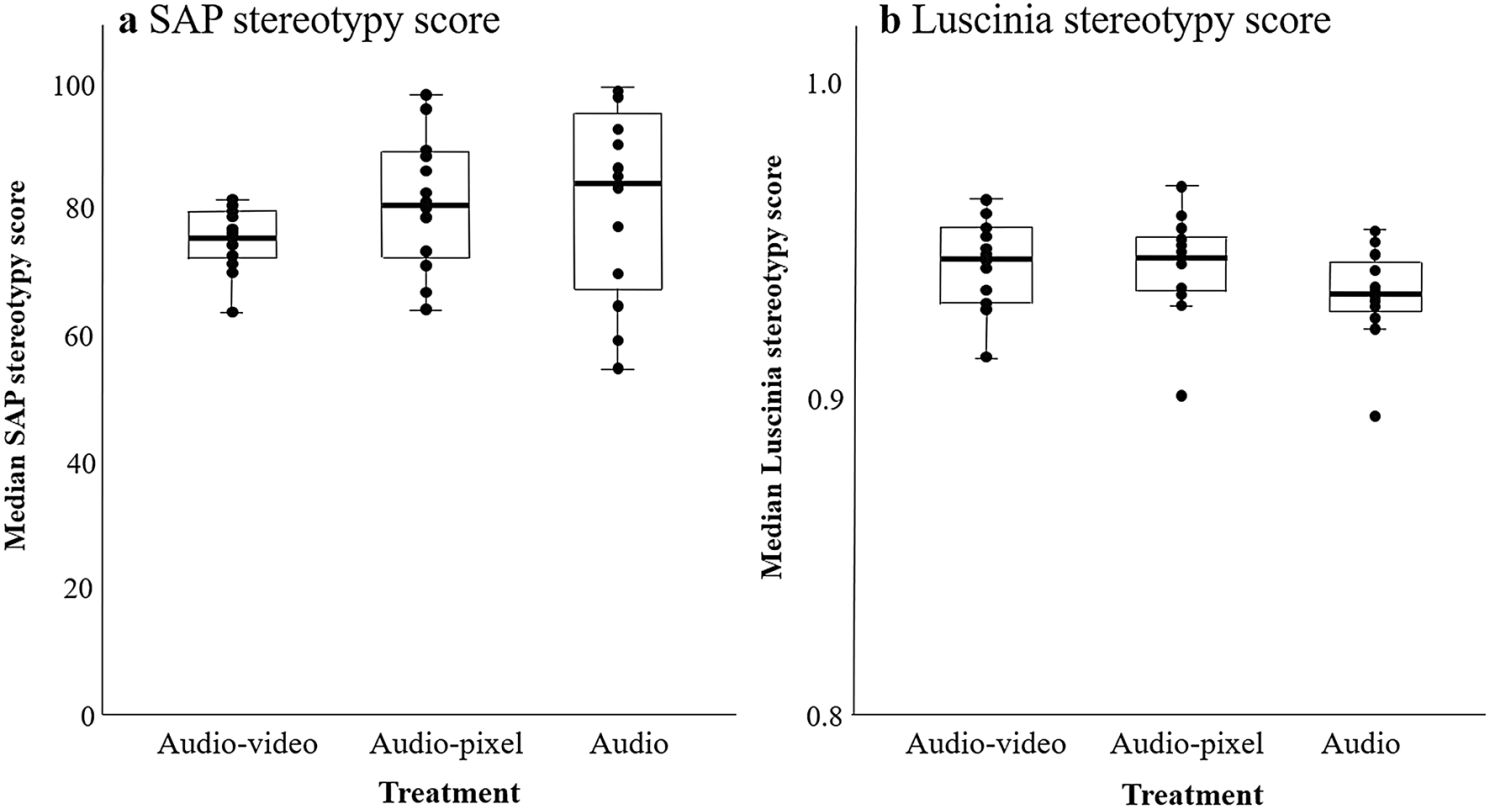
**a** SAP and **b** Luscinia stereotypy scores for the 10 randomly selected tutee motifs

**Fig. 8:**
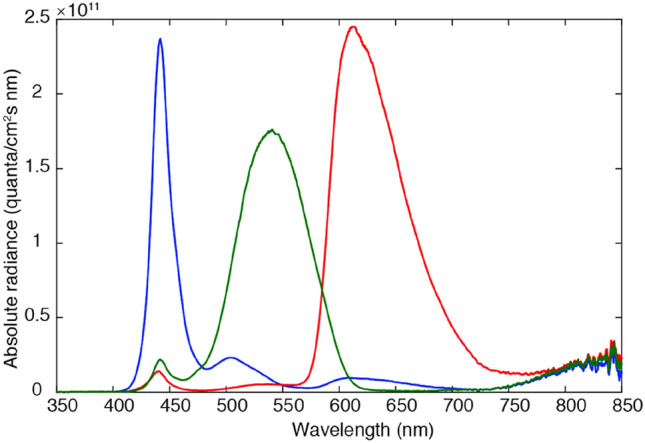
Absolute radiance of the ASUS gaming monitors used for the stimuli presentation

**Fig. 9:**
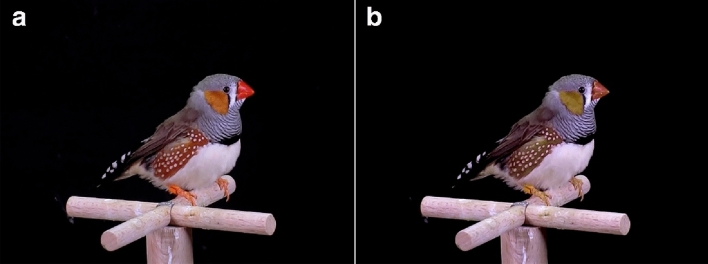
Example frames from a video stimulus. **a** Original frame before colour adjustment. **b** Colour adjusted frame which was used for stimulus presentation. Note that the colours were adjusted for presentation on a particular screen (VG248QE, ASUS, Taipei, Taiwan) and that colours might deviate if shown on a different screen or in a printed version

**Fig. 10:**
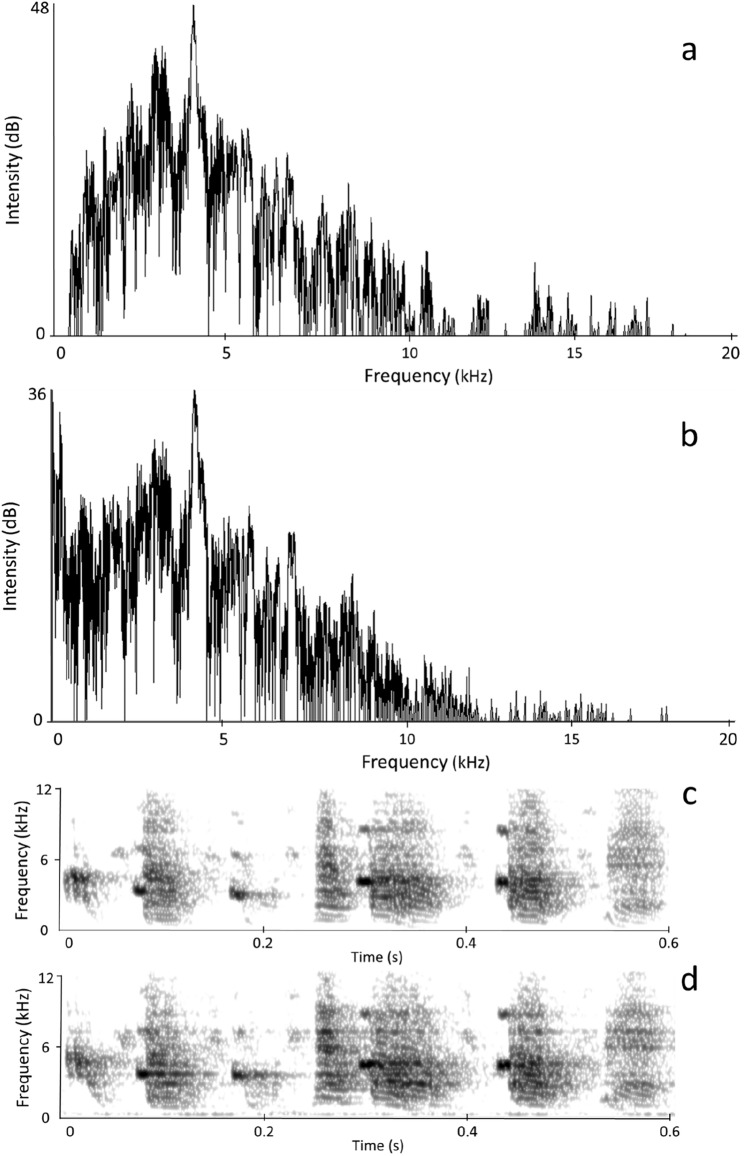
Power spectra of one motif of one of the tutors in the original recording (**a**) and re-recorded after playback in the experimental set-up (**b**). Spectrogram of the same original recording (**c**) and the re-recorded playback in the experimental set-up (**d**)

**Fig. 11:**
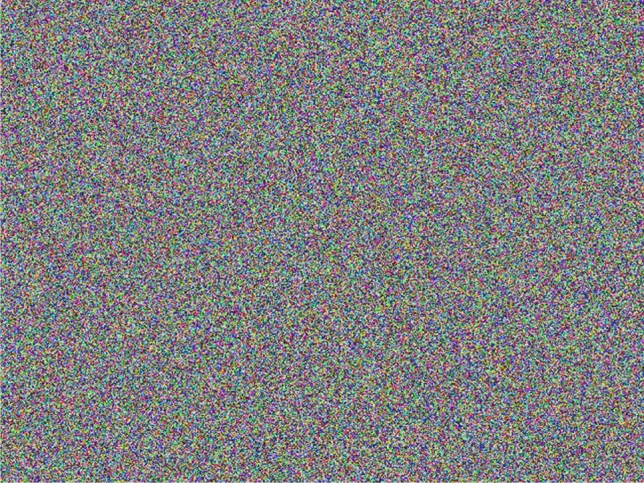
Image of the random pixels used for the displacement filter to generate the pixelated videos

To test whether birds from the different treatments differed in how stereotyped they produced their motifs, we compared the 10 randomly selected tutee motifs to each other in SAP and Luscinia. There was no difference between the tutees from the different treatment groups in the SAP or Luscinia stereotypy scores (model including ‘treatment’ was not significantly better than null model for the SAP stereotypy score (*N* = 42, *χ*^2^ = 4.36, *p* = 0.11, see Fig. 7A, Table 10A) or the Luscinia similarity score (*N* = 42, *χ*^2^ = 1.37, *p* = 0.50, see Fig. 7B, Table 10B). There was no difference between the birds raised with the different tutor song presentation schedules in the Luscinia stereotypy scores (model including ‘schedule’ was not significantly better than null model for these scores, *N* = 42, *χ*^2^ = 2.99, *p* = 0.22), but the schedules did affect the SAP stereotypy scores (model including ‘schedule’ was significantly better than null model, *N* = 42, *χ*^2^ = 14.14, *p* < 0.01). SAP stereotypy scores were higher for schedule 1 than for schedule 2 and 3.

**Table 10:** Details of best models for the (arcsine square root transformed) SAP (A) and Luscinia (B) stereotypy scores

Response variable^a^	Model term	Level	Estimate	SE	*t*
(A) SAP stereotypy score	Intercept		1.23	0.04	30.15
	Schedule				
		*Schedule 2*	− 0.15	0.05	− 3.30
		*Schedule 3*	− 0.23	0.06	− 4.09
(B) Luscinia stereotypy score	Intercept		1.32	0.005	249.4

^a^LMMs with random factor ‘Tutor group’

## Conclusion and discussion

Multimodality can enhance stimulus salience, for instance because of an alerting function of one of its components or because components in different modalities interact and affect how they are perceived (Chen and Vroomen 2013; Feenders et al. 2017; Partan and Marler 1999; Rowe 1999). Visual speech and song production cues alike might facilitate vocal learning (Kuhl and Meltzoff 1982; Slater et al. 1988; Beecher and Burt 2004; Teinonen et al. 2008; Derégnaucourt 2011; Lewkowicz and Hansen-Tift 2012; Tenenbaum et al. 2015). The aim of this study was to test whether visual exposure to a singing tutor through a high-quality video coupled with audio playback of the song has a facilitating effect on zebra finch song learning. Birds were tutored in three different conditions; audio only, audio with a video of the tutor and audio with a pixelated and reversed video. Song learning success was assessed when the juveniles had reached adulthood, using human observer visual spectrogram scoring and two automated song similarity assessment methods. We hypothesized that an auditory stimulus with concurrent visual stimulation would improve song learning compared to a unimodal auditory stimulus. Behavioural observations of the young birds showed that their engagement with the stimuli was highest in the condition where song presentation was combined with a tutor video. However, when looking at the learning outcomes, contrary to our expectations, the colour-realistic video of a singing conspecific, albeit the most attractive stimulus for the tutees, did not show improved song learning compared to the birds that received audio-only playback in any of the song similarity assessment methods.

Our prediction that visual exposure to a singing tutor improves vocal learning arose from empirical and theoretical evidence in the literature (van Kampen and Bolhuis 1991, 1993; Adret 1992; Hultsch et al. 1999; Rowe 1999). The puzzling results found in this study raise two possibilities—either our design or our assumptions were inappropriate. We will first discuss which methodological confounds can be excluded and then the wider implications of these findings regarding video tutoring.

Could it be that song learning success in this study was not affected by the visual stimulus due to the video being of insufficient video quality? Owing to technical and theoretical advancements, our study improved on potential technical shortfalls of earlier video tutoring studies such as unrealistic colours, too slow refresh rates or poor sound quality that have been a worry for animal studies in general (Oliveira et al. 2000; Ware et al. 2015; Chouinard-Thuly et al. 2017), and for an earlier video tutoring study in this species (Adret 1997). Here, we adapted our videos to the specific colour vision and flicker-fusion frequency of the zebra finch visual system, using colour-realistic imagery (Tedore and Johnsen 2017), high-speed cameras and monitors with high refresh rates. However, while this meant state-of-the-art stimulus preparation, video recordings and playbacks (other than high-quality audio playbacks) run risk of artefacts as they are not playbacks of the original stimuli, but only emulate those stimulus properties triggering the percepts associated with particular stimuli. Besides, even though we used the highest current standards, there could still be other video properties, such as deviations from real birds’ visual appearances in brightness, interference from electromagnetic fields (Pinzon-Rodriguez and Muheim 2017) or artefacts arising from the conditions during filming the singing tutors (e.g. the choice of background colour or filming the singing tutors through a layer of Plexiglas). It is also possible that the distance between the screen and the loudspeaker affected whether the birds perceived the auditory and visual stimulation as originating from the same location, which might have negatively affected potential facilitating effects of the visual stimulation on vocal learning. Any of the above or other reasons unknown to us, might have negatively affected the birds’ acceptance of the videos as a conspecific tutor. However, the behavioural data show that the birds were attracted to the videos and that they did discriminate the animated conspecific from the pixelated abstract animation: during song presentations tutees spent substantially more time close to the stimulus showing the singing male than the video showing the same bird animation but pixelated and reversed. Tutees not only used the perch near the video with the singing male more than the other perches, but they also actively flew more to the screen than tutees exposed to the pixelated video. In this context, it is important to note that the pixelated video differed from the normal tutor video in at least two aspects: the pixels were randomized and the frames were presented in reversed order. We therefore cannot tell whether the difference in tutee behaviour in response to the pixelated compared to the normal tutor videos resulted from the lack of synchrony between auditory and visual stimulation or from the lack of seeing a conspecific bird on the screen in the pixelated videos. Without being able to pin down the exact mechanism, we can state from the behavioural data that the tutor video was attractive to the birds and that they were interested in it. These observations also suggest that pairing an interesting moving visual stimulus with auditory song exposure does not necessarily lead to improved song learning. A similar observation was made by Houx and ten Cate (1999b): zebra finch tutees spent more time on the perch next to a visual stimulus in form of a taxidermic mount of an adult male zebra finch during than before its exposure. The visual stimulus, however, did not affect song learning success.

Song exposure frequency remains another debated influence on song learning (Chen et al. 2016; Derégnaucourt et al. 2013; Tchernichovski et al. 1999). In our experiment, exposure frequency varied between the different schedules used for different tutor groups, but it was always the same for the three treatments within one tutor group. This therefore seems unlikely to have systematically biased the outcome concerning the differences between treatment groups unless a ceiling or floor effect had masked treatment effects. This does not seem very probable given that there were three different tutor song presentation schedules with pronounced differences in song exposure frequencies. These ranged from 30 to 192 tutor song presentations daily which is comparable with previous playback studies where some have used comparably low song exposure frequencies and still showed some learning from the song playback (20 songs daily: Derégnaucourt et al. 2013; Funabiki and Funabiki 2009 and 40 songs daily in the operant playback study first reporting a potential negative effect of overexposure: Tchernichovski et al. 1999). Besides, the similarity scores obtained by all three similarity assessment methods did not differ between the tutoring schedules. Only two song parameters (sequence linearity and stereotypy assessed by Sound Analysis Pro) differed between the tutoring schedules. These two parameters are both related to how stereotyped a tutee produces its motifs and were lower in the schedules with more daily song exposure. This finding might support the hypothesis that a low song exposure frequency can have positive effects on song learning outcomes in zebra finch tutees (Chen et al. 2016; Tchernichovski et al. 1999).

It is always possible that our song analysis methods did not pick up any subtle difference in song learning. However, because we wanted to be able to compare our data with old and recent song learning studies, we used the three most common and established similarity assessment methods: human observers, SAP and Luscinia (and to the best of our knowledge, these three methods have not previously been used on the same data set). The overall main result that the audio–visually tutored birds did not show improved song learning was the same for all three methods. Perhaps not surprisingly, given the differences in how bioacoustic parameters are weighed in the different approaches, the three methods differed in which between group differences they detected. Most likely, the different algorithms used by the automated methods for calculating similarity picked up different parameters of song similarity than human observers assessing visual representations of the sounds. Owing to human visual perceptions principles, humans will have recognised shared patterns rather than single parameters. We used ten randomly selected motifs per tutee to calculate similarity with the automated methods SAP and Luscinia, but used only one full motif per tutee for the human observer method, which might explain why we here found a lower correlation between each automated method and the human observers than has previously been found (Luscinia: Lachlan et al. 2010; SAP: Tchernichovski et al. 2000). However, we also found a low correlation between SAP and Luscinia although these scores were based on exactly the same 10 motifs per individual. The differences between the three methods clearly deserve further attention. Note, however, that both automated methods were validated using visual scoring by human observers and that visual scoring is considered an objective suitable method for assessing song similarity as long as multiple independent observers blind to the expected outcome of the comparisons are used as judges (Jones et al. 2001). Regarding the test of our main hypothesis that audio–visual exposure should improve song learning, the similarity scores of all three methods did not show such an effect: they were never significantly higher in the audio–video group than in the audio-pixel or audio group despite the higher engagement the tutees showed with these stimuli.

A possible interpretation of these findings is therefore that multimodal stimulus presentation might increase tutee’s attention during presentation, but might not affect zebra finch song learning success. Previous studies have, however, demonstrated increased learning of an audio signal in birds when it was paired with visual stimulation (Hultsch et al. 1999; van Kampen and Bolhuis 1991, 1993), despite the use of a less naturalistic visual stimulus than in our study and several earlier ones (Bolhuis et al. 1999; Houx and ten Cate 1999b). Perhaps the sudden appearance of a social stimulus captured the attention of the zebra finch tutees in a different way than a non-specific movement and that and/or the scramble competition between the male and female juvenile we sometimes saw for the positions on perch 1 distracted them from the auditory stimulus. As demonstrated by the behavioural observations, males and females were equally attracted to the visual stimuli. It might be that the excitement of the companion by the visual social stimulation was more salient to the male tutees than the auditory song stimulus. This might also explain why the birds raised with the pixelated video had higher SAP tutor–tutee similarity scores than the birds raised with the tutor video, as both young birds seemed more excited by the tutor video than by the pixelated video (i.e. spending more time close to it and approaching it more). The pixelated video was probably less socially meaningful to the tutees than the tutor video. In future studies, we would have to test if other stimulus presentation schemes, e.g. more ongoing visual stimulus exposure instead of only very limited (sudden) exposure may lead to better song learning performance. It is also possible that the young females influenced males’ song development by reinforcing particular song structures or encouraging a particular singing style or practicing (Jones and Slater 1993; Kojima and Doupe 2011; Ruploh et al. 2013; Carouso-Peck and Goldstein 2019). Female zebra finches do not sing themselves, but in mixed-age social rearing, they normally develop socially learned song preferences for the adult male song(s) they are exposed to as sub-adults (Miller 1979; Clayton 1988; Riebel 2000, 2003; Riebel et al. 2002; Holveck and Riebel 2014). Females could have learned from the tutor and then ‘coached’ the male tutees. If they learned equally well from the different tutoring methods, they might thereby have reduced the difference between treatment groups. However, if females, like the males in this study, learned rather poorly from the model, they might have learned from their male peers instead (as documented in Honarmand et al. 2015), and in turn reinforced aspects of their peers’ songs. Through iteration of this process, both female preference and male song might have moved further away from the model song. Much will depend on how uni- versus multimodal tutoring affects female preference learning. We are not aware of any study directly investigating this question (but see Holveck and Riebel 2014, for demonstrating that live and tape-tutored females develop preferences based on early song experiences). Whether song preference learning is differentially affected by multi-compared to unimodal tutoring will thus have to be explored further in the future. Even with the careful control of the stimulus preparations, it remains possible that the filming context of the videos was suboptimal. We presented audio and video stimuli of tutors recorded when alone and singing undirected song. Zebra finch adults can, however, produce pupil-directed song towards juvenile conspecifics, which differs from undirected and adult female-directed song in several acoustic parameters (Chen et al. 2016). As female-directed and undirected song also differ in the accompanying body movements (Sossinka and Böhner 1980), it is possible (but to our knowledge not yet tested) that specific visual components proceed, accompany or follow the production of pupil-directed song and that therefore tutoring with audio or audio–visual pupil-directed song might lead to better song learning outcomes compared to tutoring with undirected song. It would be interesting to repeat the current experiment using videos of tutors producing pupil-directed song to test this idea.

It is, however, also important to stress that although video playback can provide audio–visual stimulation, it remains to be seen whether a 2-dimensional tutor can ever replace a 3-dimensional live bird, as a video provides no depth and this might mean that a substantial part of the singing movements are not visible to the bird. It is also possible that not the multimodal cues per se but the social and interactive qualities of a live tutor need to be emulated in such a setup. For instance, operant tape-tutoring, where song playback is contingent on specific tutee behaviour, can lead to better learning outcomes than passive tape-tutoring, where tutees cannot predict when song playback will occur (Adret 1993; Derégnaucourt et al. 2013, but see Houx and ten Cate 1999b). Besides, behaviour or stimuli contingent on immature song production can positively affect song learning outcomes (Carouso-Peck and Goldstein 2019; Carouso-Peck et al. 2020). With respect to the role of behaviour and social interactions as important drivers for learning to take place, there are clear parallels between song learning and imprinting processes. For zebra finches, it has been shown that mere visual exposure to a stuffed bird (which might be compared to exposure to audio-only playback), or even exposure to a live bird behind a wire had no or limited effect on being used as a model for sexual imprinting compared to when behavioural interactions could occur (ten Cate 1984; ten Cate et al. 1984). In a filial imprinting experiment, quail chicks exposed to a live hen behind a transparent screen developed a strong filial attachment, much stronger than chicks exposed to a moving stuffed hen, while exposure to a non-moving stuffed hen did not result in a measurable attachment (ten Cate 1989). Follow-up studies using animated three-dimensional visual stimulation, for instance in a virtual reality context or using robots, are necessary to further investigate the effect of presenting song production-related visual cues in addition to passive playback of tutor song on song learning as a first step and comparing such stimulation in interactive versus a non-responsive mode as a subsequent step.

In conclusion, in this study, although young birds were more attracted to and spent more time engaging with the audio–visual than the audio-only tutors, video presented visual cues related to sound production did not show a facilitating effect on vocal learning in zebra finches. Future studies with methodological adaptations are necessary to further investigate the influence of meaningful visual cues on the vocal learning process.

### Electronic supplementary material

Below is the link to the electronic supplementary material.

Supplementary file1 (MP4 35538 KB) **Online Resource 1 **Example of one colour-adjusted stimulus video used for tutoring in the Audio-video condition.

Supplementary file2 (MP4 38839 KB) **Online Resource 2 **Example of one colour-adjusted stimulus video used for tutoring in the Audio-pixel condition.

## Data Availability

The datasets generated and analysed during the current study will be available in the Dryad repository.

## Appendix

Output of the MATLAB scrip provided by Tedore and Johnson (2017) with the correction factors (new calculations with ASUS adjustments).

**Table 11:** Results for post-hoc comparison of proportion of time that tutees spent in different areas of the cage (corrected for the perch length in that area)

Contrast	Estimate	SE	*t*	*p*
Area 1 audio vs Area 1 audio–video	− 0.32	0.05	− 6.72	**< 0.01**
Area 1 audio vs Area 1 audio-pixel	− 0.14	0.05	− 2.95	0.09
Area 1 audio vs Area 2 audio	0.07	0.05	1.56	0.82
Area 1 audio vs Area 2 audio–video	0.27	0.05	5.60	**< 0.01**
Area 1 audio vs Area 2 audio-pixel	0.16	0.05	3.33	**0.03**
Area 1 audio vs Area 3 audio	0.17	0.05	3.61	**0.01**
Area 1 audio vs Area 3 audio–video	0.36	0.05	7.47	**< 0.01**
Area 1 audio vs Area 3 audio-pixel	0.24	0.05	5.13	**< 0.01**
Area 1 audio–video vs Area 1 audio-pixel	0.18	0.05	3.77	**< 0.01**
Area 1 audio–video vs Area 2 audio	0.40	0.05	8.28	**< 0.01**
Area 1 audio–video vs Area 2 audio–video	0.59	0.05	12.32	**< 0.01**
Area 1 audio–video vs Area 2 audio-pixel	0.48	0.05	10.05	**< 0.01**
Area 1 audio–video vs Area 3 audio	0.49	0.05	10.33	**< 0.01**
Area 1 audio–video vs Area 3 audio–video	0.68	0.05	14.19	**< 0.01**
Area 1 audio–video vs Area 3 audio-pixel	0.57	0.05	11.85	**< 0.01**
Area 1 audio-pixel vs Area 2 audio	0.22	0.05	4.51	**< 0.01**
Area 1 audio-pixel vs Area 2 audio–video	0.41	0.05	8.55	**< 0.01**
Area 1 audio-pixel vs Area 2 audio-pixel	0.30	0.05	6.28	**< 0.01**
Area 1 audio-pixel vs Area 3 audio	0.31	0.05	6.56	**< 0.01**
Area 1 audio-pixel vs Area 3 audio–video	0.50	0.05	10.42	**< 0.01**
Area 1 audio-pixel vs Area 3 audio-pixel	0.39	0.05	8.08	**< 0.01**
Area 2 audio vs Area 2 audio–video	0.19	0.05	4.04	**< 0.01**
Area 2 audio vs Area 2 audio-pixel	0.08	0.05	1.77	0.70
Area 2 audio vs Area 3 audio	0.10	0.05	2.05	0.51
Area 2 audio vs Area 3 audio–video	0.28	0.05	5.90	**< 0.01**
Area 2 audio vs Area 3 audio-pixel	0.17	0.05	3.57	**0.02**
Area 2 audio–video vs Area 2 audio-pixel	− 0.11	0.05	− 2.27	0.37
Area 2 audio–video vs Area 3 audio	− 0.09	0.05	− 1.98	0.56
Area 2 audio–video vs Area 3 audio–video	0.09	0.05	1.87	0.64
Area 2 audio–video vs Area 3 audio-pixel	− 0.02	0.05	− 0.46	0.99
Area 2 audio-pixel vs Area 3 audio	0.01	0.05	0.28	1.00
Area 2 audio-pixel vs Area 3 audio–video	0.20	0.05	4.14	**< 0.01**
Area 2 audio-pixel vs Area 3 audio-pixel	0.09	0.05	1.80	0.68
Area 3 audio vs area 3 audio–video	0.18	0.05	3.85	**< 0.01**
Area 3 audio vs area 3 audio-pixel	0.07	0.05	1.52	0.84
Area 3 audio–video vs Area 3 audio-pixel	− 0.11	0.05	− 2.33	0.33

Significant *p*-values are indicated in bold
